# Biofilm morphology and antibiotic susceptibility of methicillin-resistant *Staphylococcus aureus* (MRSA) on poly-D,L-lactide-*co*-poly(ethylene glycol) (PDLLA-PEG) coated titanium

**DOI:** 10.1016/j.bioflm.2024.100228

**Published:** 2024-10-05

**Authors:** Adam Benedict Turner, David Zermeño-Pérez, Margaritha M. Mysior, Paula Milena Giraldo-Osorno, Begoña García, Elizabeth O'Gorman, Shafik Oubihi, Jeremy C. Simpson, Iñigo Lasa, Tadhg Ó Cróinín, Margarita Trobos

**Affiliations:** a Department of Biomaterials, Institute of Clinical Sciences, Sahlgrenska Academy, University of Gothenburg, Gothenburg, Sweden; b Centre for Antibiotic Resistance Research in Gothenburg (CARe), Gothenburg, Sweden; c Ashland Specialties Ireland Ltd., Mullingar, Ireland; d School of Biomolecular and Biomedical Science, University College Dublin, Dublin, Ireland; e Cell Screening Laboratory, UCD School of Biology & Environmental Science, University College Dublin, Dublin, Ireland; f Microbial Pathogenesis Laboratory. Navarrabiomed-Complejo Hospitalario de Navarra (CHN)-Universidad Pública de Navarra (UPNA), IDISNA, Pamplona, Navarra, Spain

**Keywords:** Poly-D,L-lactide **(**PDLLA), Polyethylene-glycol (PEG), biofilm, Methicillin-resistant *Staphylococcus aureus* (MRSA), Antibiotics

## Abstract

Biodegradable polymeric coatings are being explored as a preventive strategy for orthopaedic device-related infection. In this study, titanium surfaces (Ti) were coated with poly-D,L-lactide (PDLLA, (P)), polyethylene-glycol poly-D,L-lactide **(**PEGylated-PDLLA, (PP20)), or multi-layered PEGylated-PDLLA (M), with or without 1 % silver sulfadiazine. The aim was to evaluate their cytocompatibility, resistance to *Staphylococcus aureus* biofilm formation, and their potential to enhance the susceptibility of any biofilm formed to antibiotics. Using automated high-content screening confocal microscopy, biofilm formation of a clinical methicillin-resistant *Staphylococcus aureus* (MRSA) isolate expressing GFP was quantified, along with isogenic mutants that were unable to form polysaccharidic or proteinaceous biofilm matrices. The results showed that PEGylated-PDLLA coatings exhibited significant antibiofilm properties, with M showing the highest effect. This inhibitory effect was stronger in *S. aureus* biofilms with a matrix composed of proteins compared to those with an exopolysaccharide (PIA) biofilm matrix. Our data suggest that the antibiofilm effect may have been due to (i) inhibition of the initial attachment through microbial surface components recognising adhesive matrix molecules (MSCRAMMs), since PEG reduces protein surface adsorption via surface hydration layer and steric repulsion; and (ii) mechanical disaggregation and dispersal of microcolonies due to the bioresorbable/degradable nature of the polymers, which undergo hydration and hydrolysis over time. The disruption of biofilm morphology by the PDLLA-PEG co-polymers increased *S. aureus* susceptibility to antibiotics like rifampicin and fusidic acid. Adding 1 % AgSD provided additional early bactericidal effects on both biofilm and planktonic *S. aureus*. Additionally, the coatings were cytocompatible with immune cells, indicating their potential to enhance bacterial clearance and reduce bacterial colonisation of titanium-based orthopaedic biomaterials.

## Highlights

• PEGylated-PDLLA coatings had antibiofilm effect against multiresistant *S. aureus* and were cytocompatible.
• These coatings may inhibit bacterial surface adhesins and mechanically disaggregate biofilms, increasing *S. aureus* susceptibility to antibiotics.
• Incorporation of silver into the coatings provided additional bactericidal effects.
• PEGylated-PDLLA coatings can potencially enhance the infection resistance of titanium.
• HCS-confocal microscopy provides unbiased automated 3D-image analysis of *S. aureus* biofilms on biomaterials.

## Graphical abstract

**Figure:**
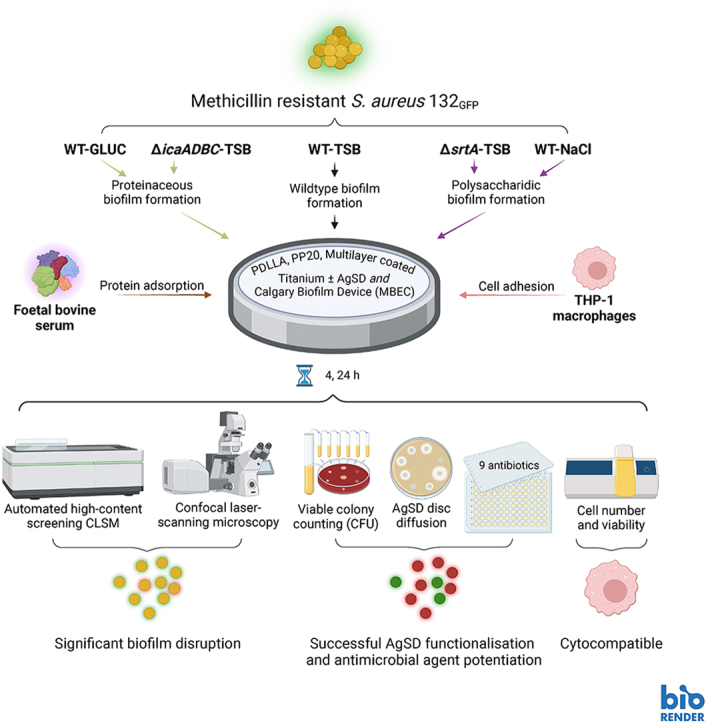

## 1 Introduction

Orthopaedic device-related infection (ODRI) is one of the most significant threats to the successful implantation and integration of orthopaedic implants. It represents the most severe complication associated with the use of implanted medical devices with complicated and costly treatment options, impacting the patient's quality of life. The risk of infection varies depending on the implant type and location, ranging from 1 to 2 % for hip and knee prostheses [[1], [2], [3]], but up to 30 % for open fracture fixation devices [4]. The occurrence of reinfection/infection relapse has been reported to be as high as 40 % [1,2,5,6].

Contaminating bacterial species begin the process of infection by adhering to the surface of the orthopaedic device and forming a biofilm, from which they can multiply and propagate [7]. In the case of ODRI, the most common and significant aetiological agents are staphylococci, of which *Staphylococcus aureus* often poses the greatest threat to the patient [8,9].

*S. aureus* initiates attachment to an implanted biomaterial through the action of a family of microbial surface components recognising adhesive matrix molecules (MSCRAMMs) [10]. These proteins recognise and interact with host blood and coagulation proteins that adsorb to the medical device immediately after implantation [11]. Many of these MSCRAMMs share a common C-terminal amino acid sorting motif (LPXTG), which is responsible for their anchorage to the cell wall following cleavage by Sortase A (SrtA) [12]. Once irreversibly attached to a material surface, *S. aureus* begins to form a biofilm, a community of bacterial cells in a self-produced extracellular matrix consisting of proteins, extracellular DNA (eDNA), and polysaccharides [13,14]. The role of surface proteins is not limited to facilitating primary adhesion, they can also contribute to the formation of the extracellular matrix in which the bacteria are embedded. The biofilm matrix produced by *S. aureus* can be either proteinaceous or polysaccharide in nature. The proteinaceous matrix can be formed by various proteins including Bap, SasG, FnbBP or Protein A. In contrast, the polysaccharide biofilm is formed by a polymer of poly-β(1–6)N-acetylglucosamine (PIA/PNAG) whose synthesis depends on the *icaADBC* operon [20,21]. Some strains of *S. aureus* produce a proteinaceous matrix, while others produce a polysaccharidic matrix, and some strains can alternate between these two types of matrices depending on environmental conditions [16]. It is unclear whether the colonisation behavior of *S. aureus* strains on implants varies based on the nature of their biofilm matrix. The combination of altered gene expression, protein production, and metabolic activity during the biofilm lifestyle creates a microenvironment highly resilient to the host immune system and antibiotic treatment [15]. Both physical and metabolic properties contribute to antibiotic tolerance, with biofilm viscosity hindering antibiotic penetration, and altered gene expression rendering common antibiotic targets ineffective [16]. High bacterial cell densities found in biofilm also enable conjugative transfer of resistance carrying plasmids – further threatening the use of antibiotics in future infectious disease treatment [[17], [18], [19]].

Orthopaedic device implantation is increasing and expected to increase further due to improvements in surgical practice and an aging population [3]. As the number of these procedures increases, the incidence of related infection is also likely to increase. Therefore, developing and investigating novel strategies to prevent orthopaedic device infections is crucial. Anti-infective strategies are typically categorized as either antimicrobial or antifouling based on their mechanisms of action [20]. Antimicrobial strategies aim to kill infecting microorganisms at, or surrounding the implant site through various methods such as topographical modifications [21], incorporation of bactericidal metals into an implant alloy [22], or functionalisation with antimicrobial peptides or compounds [23]. Antifouling strategies, on the other hand, do not kill bacteria but prevent biofilm formation or repel microbial adhesion entirely. These strategies usually involve enzyme/signal-based, superhydrophobic, zwitterionic, or polyethylene-glycol (PEG) based coatings [24].

PEG-based coatings are the most frequently utilised, as the production process is relatively cost-effective, scalable, and can be applied to the large range of topographies presented by orthopaedic devices [25]. The antifouling properties of PEG-based coatings are thought to result from the formation of a hydration layer and steric hindrance limiting the adsorption of host proteins and contaminating bacterial species alike [24,25]. PEG absorbs water to create a tightly bound hydration layer, making the surface thermodynamically unfavourable for microbial adhesion. Additionally, the compression of PEG chains produces electrostatic repulsion, resulting in a similarly unattractive surface for adhesion [24].

These coatings are also of interest for preventing ODRI as they are non-toxic, bioresorbable *in vivo*, and possess highly customisable resorption kinetics [26,27]. By introducing co-polymers and modifying PEG chain length, the duration of antifouling properties can be increased or decreased to meet the needs of different implant applications [27]. Moreover, PEG-based coatings can be functionalised/blended with different antimicrobial agents depending on their chemistries [27]. This presents the opportunity to design highly customisable, time-releasing, antifouling and antimicrobial devices that prevent per-implantation infection, while maintaining successful host integration of titanium implants.

In this study, we investigate a PEGylated poly-D,l-lactide (PDLLA) coating with increasing PEG content on titanium with the following objectives: (i) to evaluate its cytocompatibility with immune cells; (ii) to assess its anti-adhesion effect against methicillin-resistant *S. aureus* (MRSA); (iii) to determine the coating's effect based on the composition of the biofilm matrix (proteinaceous or polysaccharide); (iv) to analyse its impact on biofilm morphology; and (v) to evaluate whether these coatings alter the susceptibility of MRSA biofilms to clinically relevant antimicrobial agents.

## 2 Methods

### 2.1 Polymer synthesis

All polymers used were produced by Ashland Specialties Ireland. The ring opening polymerization (ROP) in bulk of poly D,L-lactide (PDLLA) (P), poly D,L-lactide-co-methyl ether poly (ethylene glycol) (PDLLA-PEG 80:20) (PP20) and poly D,L-lactide-co-methyl ether poly (ethylene glycol) (PDLLA-PEG 60:40) (PP40) was carried out by adding the dried monomer, D*,L*-lactide; initiator, mPEG_5000_ and catalyst, tin (II) 2-ethylhexanoate inside a stainless-steel reactor. The reactions were carried out under an oxygen free environment at 130–150 °C for 5–8 h, and samples were taken hourly for proton nuclear magnetic resonance (H NMR) and Gel permeation chromatography (GPC) kinetics analyses [27].

### 2.2 Biomaterial preparation

#### 2.2.1 Spin coated titanium (Ti) samples

Ti6Al4V (Grade V) coupons were cleaned and sterilised before the application of the polymer coatings. Polymer solutions were made at a concentration of 20 % wt./vol in chloroform. Spin coating was then performed by depositing 75 μL of the polymer solutions to homogeneously cover the entire surface area of the discs (78.50 ± 0.05 cm^2^). A rotational speed of 6000 RPM was used for 30 s for each layer. Samples were left to dry overnight before testing.

**Fig. 1:**
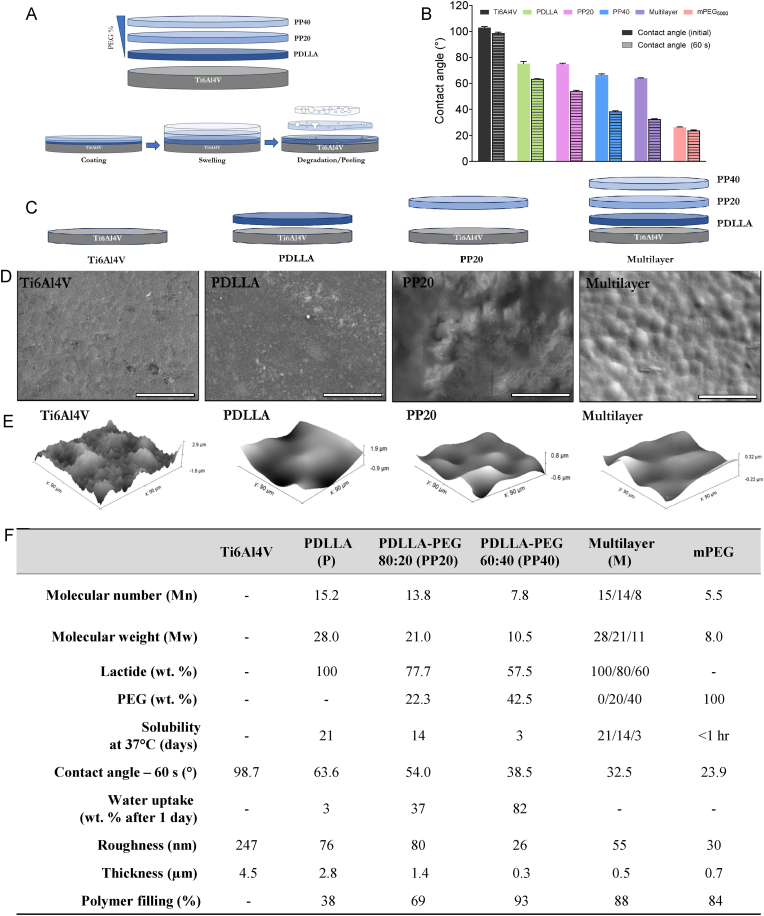
Material and coating characterisation. **A)** Coating schematic and PEG % gradient for bioresorbable polymer coating strategy. **B)** Contact angle measurements over time between 0 and 60 s. **C)** Schematic with the coating compositions for each polymer coating group. **D)** SEM micrographs of material surfaces. **E)** AFM topographical analyses. **F)** Summary table of material characterisation for Ti6Al4V, homopolymer and copolymers in monolayer and multilayer. Scale bar = 200 μm. **Abbreviations:** Polyethylene glycol (PEG), Poly-D,l-lactic acid (PDLLA), PDLLA-PEG 80:20 (PP20), PDLLA-PEG 60:40 (PP40), Multilayer (M).

Due to the rapid solubilisation of PP40 and early detachment from Ti, it was not evaluated as a monolayer. Therefore, a multilayer gradient coating was designed from the polymers used, where Ti was first coated with PDLLA due to its closer affinity to Ti6Al4V. A secondary and tertiary layer were then applied using PP20 and PP40, respectively, (Fig. 1A–C) enabling the study of the PP40. The tri-layer coating is referred to as the multilayer for the purposes of this article.

#### 2.2.2 Calgary biofilm device peg-lid dip coating

To coat the pegged lids of a Calgary Biofilm Device (Innovotech, Edmonton, Canada), 250 μL of the polymer solutions (20 % wt./vol) were multichannel-pipetted into each well of a 96-well plate. The Calgary Biofilm Device lid was then submerged manually for 10 s, guaranteeing that each peg was immersed in the solution during dip coating, followed by slowly raising the lid to allow evaporation of the solution over 10 s. The procedure was carried out three times, followed by inversion of the lid to leave the coated pegs facing upwards. For the M coating, the Calgary Biofilm Device lid was first coated with the PDLLA, followed by the PP20 and finally PP40. The dip coated pegs were left to dry in a fume hood for 24 h before incubation.

#### 2.2.3 Silver sulfadiazine loading

Silver sulfadiazine (AgSD) was added in powder form at 0.5–5.0 % wt./vol. to the polymer solutions previously prepared at 20 % wt./vol. Solutions were thoroughly vortexed to homogenise the suspension before application as a coating following the previously described spin coating procedure on titanium (Ti) samples.

### 2.3 Material characterisation

#### 2.3.1 Water contact angle

Contact angle measurements were performed to characterise the degree of wettability of the polymeric coatings on Ti6Al4V discs. The measurements were performed by static contact angle using deionized water (10 μL) as solvent. The goniometer (Ossila, Netherlands) carried out video recordings for 60 s to evaluate the change in contact angle over time. The results were processed by edge detection using the Ossila Contact Angle 4.1 software.

#### 2.3.2 Scanning Electron Microscopy (SEM)

Scanning Electron Microscopy (SEM) was conducted using a JEOL IT200 operating at an accelerating voltage between 10 and 15 kV. Images were taken in different locations at 200 × magnification to visualise topography and homogeneity of the coatings. Titanium discs spin-coated with polymers were previously dried for 24 h in a vacuum desiccator and mounted on carbon tape. Polymers were able to be visualised without a conductive coating.

#### 2.3.3 Topographical analyses

Atomic force microscopy (AFM) was carried out to analyse the surface topographies using amplitude modulation AFM (MFP-3D, Asylum Research). Si probes (NCH, Nanosensors) were used having a nominal spring constant of 42 N/m and resonance frequency of 330 kHz. Representative images at 90 × 90 μm were collected in multiple locations to analyse topography and homogeneity of coatings. Root mean square (RMS) roughness was evaluated using the entirety of the 90 × 90 μm area. Thickness was quantified by measuring the lowest and highest topographical features in the surface. Polymer filling was calculated by the decrease in thickness of the Control-Ti6Al4V after polymer coating. 3D images were flattened to remove sample tilt.

#### 2.3.4 Water uptake and solubility

Polymer discs (10 mm diameter and 1 mm thickness) were prepared by melt casting using a silicone mould. Each well was filled with 500 ± 5 mg of polymer granules. The melting procedure was performed under vacuum (−5 mPa) at 110 °C for 30 min. After solidification, samples were stored under vacuum in a desiccator. Samples were incubated at 37 °C in 10 mL PBS (pH 7.2) and 100 rpm. Water uptake was calculated by the change in weight from the initial dry mass and final wet mass after 24 h. Samples continued to be incubated until complete solubilisation was achieved and no solid or precipitates were present.

### 2.4 Construction of Sortase A mutant

To construct the Sortase A mutant (Δ*srtA*) the plasmid pMAD*srtA*AD [28] was used. Briefly, two DNA fragments were amplified with the primer pairs BG_STAP79/LM97 and LM98/BG_STAP86 from the *S. aureus* MW2 strain [29]. The two PCR fragments were fused through overlapping PCR using primers BG_STAP79 and BG_STAP86, cloned into the pJET 1.2 vector (Fisher Scientific Ref. 10809720) and then subcloned into the pMAD vector [30] digested with *Nco*I and *Bam*HI, generating plasmid pMAD*srtA*AD. This plasmid was transformed into the *S. aureus* 132 [31] (purified from *S. aureus* RN4220) [32], by electroporation [33]. Homologous recombination experiments were performed as previously described [34]. Erythromycin-susceptible white colonies, which did not further contain the pMAD plasmid, were tested by PCR using the primers BG_STAP88 and BG_STAP90 and sanger sequencing was used to confirm the generated isogenic mutant. The sequences of the primers are shown in Table 1.

**Table 1:** Oligonucleotides.

Oligonucleotide	Sequence
BG_STAP79	**ccatgg**gaattaacatggttgtcttg
LM97	ctttcggaatttgaggtttacgttaaggctccttttatacatt
LM98	agccttaacgtaaacctcaaattccgaaaga
BG_STAP86	**ggatcc**tttttcccaaacgcctgtct
BG_STAP88	ggatatacgactgagggaacgtgg
BG_STAP90	ccgatttaagtgctttgccgg

Restriction enzymes sites are indicated in **bold**.

### 2.5 Construction of plasmid encoding green fluorescent protein

Plasmid pCN47*Phyper::cGFP* expressing green fluorescent protein (GFP) constitutively was constructed as follows. The DNA region containing tandem copies of the *rrnB T1* transcription terminator upstream of *Phyper* promoter, the *Hyper-SPO1* (*Phyper*) constitutive promoter, and the *hly* 5′ UTR sequence fused with *gfpmut2* gene was purified by digestion of the pAD1-cGFP plasmid [35] with *Eco*RI/*Sal*I. This insert was purified and cloned into pCN47 plasmid [36]. This plasmid was transformed into the *S. aureus* 132 [31], *S. aureus* 132 Δ*ica* [31] and *S. aureus* 132 Δ*srtA* (this study) (purified from *S. aureus* RN4220) [32], by electroporation [33]. The fluorescence of these strains was confirmed by microscopy (Leica DM4000B, Spain).

### *2.6* *S. aureus* culture conditions

The *S. aureus* strains and plasmids used in this study are summarised in Table 2.

**Table 2:** *S. aureus* strains and plasmids used in this study.

Strains and plasmids	Relevant characteristic(s)	Reference/Source
**Strains**		
*S. aureus* 132	MRSA clinical strain; biofilm positive	[31]
*S. aureus 132*_GFP_	132 transformed with *pCN47::Phyper::cGFP* plasmid	This study
*S. aureus* 132 Δ*icaADBC*_GFP_	132 with deletion of the *icaADBC* operon transformed with *pCN47::Phyper::cGFP* plasmid	This study
*S. aureus* 132 Δ*srtA*_GFP_	132 with deletion of *srtA* gene transformed with *pCN47::Phyper::cGFP* plasmid	This study
**Plasmids**		
pCN47:*Phyper::cGFP*	pCN47 expressing GFP under control of *Phyper* constitutive promoter	This study

All strains were streaked onto agar from glycerol stocks stored at - 80 °C. Different inoculum conditions were utilised depending on the experimental approach used.

*S. aureus* 132_GFP_, *S. aureus* 132Δ*ica*_GFP_, and *S. aureus* 132 Δ*srtA*_GFP_ were streaked onto tryptic soy agar (TSA; Scharlau, Barcelona, Spain) containing erythromycin (10 μg/mL) and incubated overnight at 37 °C. This strain was selected due to its well characterised ability to alternate between a proteinaceous or exopolysaccharidic biofilm matrix depending on the environmental conditions/growth media in which it is grown [31]. Three colonies were inoculated into tryptic soy broth (TSB, Scharlau, Barcelona, Spain) containing erythromycin (10 μg/mL) and further incubated overnight shaking at 200 rpm and 37 °C. This suspension was diluted in TSB, TSB supplemented with 0.25 % glucose (TSB_G_) to induce proteinaceous biofilms, or TSB supplemented with 3 % NaCl (TSB_N_) to induce polysaccharidic biofilms, to a final optical density (OD) of 0.1 at 600 nm. One mL of the inoculum (OD 0.1) was then added to the test samples (Ti, P, PP20, M) and incubated statically at 37 °C for 4 h or 24 h. Following this, either viable colony counting, or high-content confocal microscopy was performed.

For susceptibility testing of the strains, minimum inhibitory concentration (MIC) and minimum biofilm eradication concentration (MBEC), *S. aureus* 132_GFP_ was streaked onto TSA containing 10 μg/mL erythromycin and incubated overnight at 37 °C. Single colonies were selected and inoculated into cation-adjusted Müller-Hinton Broth (caMHB) or TSB, respectively, to achieve a final concentration of 1 × 10^6^ (MIC) and 5 × 10^5^ CFU/mL (MBEC), respectively, and planktonic and biofilm *S. aureus* were then challenged against an antibiotic panel as described below.

### *2.7* *S. aureus* biofilm formation on polymer-coated titanium (viable colony counting)

One mL of an *S. aureus* 132 (OD_600_ = 0.1) inoculum prepared as described above was added to 48-well plates containing polymer spin-coated Ti discs ± AgSD (PDLLA, PP20, Multi-layered). Uncoated Ti discs were used as controls. These were incubated statically at 37 °C for 4 h or 24 h. Following this, discs were gently rinsed in saline (0.9 %) to remove non-adhered *S. aureus* 132 and were transferred to a 15 mL Falcon tube containing 1 mL saline. This was sonicated for 30 s and vortexed for 1 min to detach and disaggregate the adhered biofilms. The resuspended biofilms were then tenfold serially diluted to 10^−7^ in saline and Triton-X100 (0.1 %) and 5 μL was spot plated on 5 % Columbia Horse Blood Agar (HBA, Media Department, Clinical Microbiology Laboratory, Sahlgrenska University Hospital, Sweden) for subsequent enumeration. For the AgSD containing polymers, viability of planktonic *S. aureus* 132_GFP_ was also investigated. Here, 200 μL of growth media surrounding the discs was taken and transferred to a sterile 96-well plate. This was then ten-fold serially diluted to 10^−7^ in saline and Triton-X100, and 5 μL was spotted onto HBA for subsequent enumeration.

### 2.8 High-content screening confocal microscopy

After 4 h or 24 h *S. aureus* 132_GFP_ (TSB, TSB_G_, TSB_N_), *S. aureus* 132 Δ*ica*_GFP_ (TSB) *or S. aureus* 132 Δ*srtA*_GFP_ (TSB) biofilm growth on the respective polymer-coated titanium, samples were individually rinsed by submersion in PBS to remove non-adherent bacteria. To visualise dead bacterial cells, samples were then stained with Propidium Iodide (PI, FilmTracer) in saline for 20 min protected from light. Samples were then rinsed again to remove any unbound stain, carefully inverted, transferred, and mounted in optically clear, glass-bottomed 24-well plates (MoBiTec GmbH, Austria) for imaging on the Opera Phenix High-Content Screening System (Revvity, Austria) equipped with a 63 × /1.15 NA water immersion objective. Using the in-built software well mapping grid, five equidistant fields of view were selected from each well to provide a standardised surface area for imaging and quantification of the biofilm coverage on each disc surface. The same fields of view and disc areas were imaged across all wells in a 24-well plate. Z-stacks were taken at 3 μm slices through the z-axis of the biofilm, and biofilm parameters were quantitatively analysed using the Harmony High-Content Imaging and Analysis Software (Revvity, Austria). Parameters investigated included *biofilm biovolume*, *biofilm microcolony biovolume*, and *biofilm microcolony height*. These measured all connected signal positive voxels within a 3D space (biovolume) and the highest/lowest z-plane in which signal positive pixels were detected (height). Three independent experiments (n = 3) were performed with two technical replicates.

### 2.9 Disc diffusion

To test the antimicrobial efficacy of polymers blended with 0–5 % AgSD, a standard disc diffusion test was performed based on the Kirby-Bauer test [37]. Spin coated samples blended with Silver Sulfadiazine (AgSD) at 0.5–5.0 % wt./vol. were tested against *S. aureus* 132_GFP,_ using filter paper with AgSD 1.0 % wt./vol as a positive control. Plates were incubated for 24 h, and zones of inhibitions were measured. Zone diameters were calculated using ImageJ [38], using a 10 mm disc as reference to set scale.

### 2.10 Minimum biofilm eradication concentration (MBEC)

All pegs of the lid of a standard 96-well Calgary Biofilm Device (Innovotech) were dip-coated in the respective polymers (PDLLA, PP20, M) as described above. Uncoated polystyrene peg lid plates were used as a control (PS control). A volume of 150 μL of 5 × 10^5^ CFU/mL prepared in TSB, TSB_G_ or TSB_N_ was added to each well of a 96-well Calgary Biofilm Device plate (Innovotech) and the pegs were immersed into the bacterial inoculum. Three wells in each plate were inoculated with sterile growth media as negative controls. Each plate was incubated at 125 rpm and 37 °C for 24 h to allow biofilm to form. Following primary incubation, biofilms grown on pegs were rinsed in saline to remove non-adherent bacteria. Three pegs were removed to perform viable colony counting to quantify viable biofilm bacteria, and the plate lid was transferred to a 96-well plate containing a freeze-dried custom-made antibiotic panel (SWE1GOTH, Sensititre, UK) (Table 3) re-suspended in ca-MHB and incubated statically at 37 °C for 20 h.

**Table 3:** Antibiotic panel and concentrations present in the custom made Sensititre plate (SWE1GOTH) used for this investigation [39].

Antibiotic	Concentration range (μg/mL)
Rifampicin (RIF)	0.01–128
Levofloxacin (LEV)	0.01–128
Fusidic acid (FA)	0.5–256
Clindamycin (CLI)	0.125–256
Linezolid (LZD)	2–128
Sulfamethoxazole/Trimethoprim (SXT)	1/19–62/1216
Oxacillin (OXA)	0.5–64
Vancomycin (VAN)	0.5–128
Cefoxitin (FOX)	1–64

Following secondary incubation, peg lids were rinsed twice in saline and transferred to a “recovery” plate containing ca-MHB supplemented with 50 mg/mL l-histidine, 50 mg/mL l-cysteine, and 100 mg/mL reduced glutathione. The plates were sonicated for 1 min (42 kHz) to disaggregate and release the adhered biofilms into the surrounding media. Following this, the peg lid was removed, and a new standard sterile lid was added, and the plates were incubated for a final time overnight statically at 37 °C. MBEC values were obtained by visual analysis of the first non-turbid concentration for each antimicrobial agent. Either two (n = 2, WT-TSB_G_, WT-TSB_N_, Δ*ica-*TSB, Δ*srtA-*TSB) or three independent experiments (n = 3, WT-TSB) were performed with two technical replicates, and the first non-turbid concentration observed in at least 2 of the experiments was recorded.

### 2.11 Minimum inhibitory concentration (MIC)

To characterise and confirm the MRSA phenotype of the *S. aureus* 132_GFP_ strain, 100 μL of 1 × 10^6^ CFU/mL of the inoculum in ca-MHB, prepared as described above, was added to the 96-well antibiotic panel containing 100 μL of each antibiotic in ca-MHB (Table 3). Plates were then incubated statically at 37 °C for 20 h. Following this, MIC values were obtained by visual analysis of the first non-turbid well.

### 2.12 Protein adsorption to polymer-coated Ti

Uncoated Ti and polymer-coated samples were incubated in 1 mL heat-inactivated foetal bovine serum (HI-FBS; ThermoFisher, Waltham, United States) in 24-well plates (Nunc) at 4 °C for 4 h or 24 h. Following this, each disc was gently rinsed in sterile water and transferred to a new 24-well plate and allowed to air dry under laminar flow. Sterile, clean samples without protein were used as blanks. Once dried, 200 μL of BCA reagent (1:50, CuSO4: Buffer) was added, and samples were incubated at 37 °C shaking at 150 rpm for 30 min. From this, 150 μL were transferred to a 96-well plate, and absorbance (562 nm) was measured in a plate reader (FluostarOmega, BMG LABTECH, Ortenberg, Germany). Total protein concentrations were derived from a BSA standard curve. Three independent experiments were performed (n = 3) with two technical replicates.

### 2.13 Cell viability and adhesion

THP-1 human monocytic cell line (ATCC TIB-202, Manassas, VA, USA) in passage 4 was grown in Roswell Park Memorial Institute 1640 medium (RPMI) supplemented with 10 % HI-FBS, 0.5 % β-mercaptoethanol (Sigma Aldrich, Munich, Germany) and 1 % penicillin/streptomycin solution (Gibco Life Technologies, Carlsbad, CA, USA). The cells were grown in 75 cm^2^ culture flasks at 37 °C in a humidified incubator with 5 % CO_2_. To induce macrophage differentiation, THP-1 monocytes were stimulated with 10 ng/mL phorbol-12-myristate-13-acetate (PMA, Sigma Aldrich, Munich, Germany) for 48 h, followed by 24 h of resting time in fresh RPMI without PMA. Macrophages were detached using trypsin (Gibco Life Technologies, Waltham, USA) and 5 × 10^5^ cells/mL were seeded onto Ti and polymer-coated samples ± AgSD and incubated for further 24 h at 37 °C and 5 % CO_2_.

Cell viability was analysed by quantifying the release of lactate dehydrogenase (LDH) into the cell culture medium using the CyQUANT™ LDH Cytotoxicity Assay (Thermo Fisher Scientific, Roskilde, Denmark), following the manufacturer's protocol. In brief, 50 μL of supernatant from each well was transferred to Nunc 96-well plates (Thermo Fisher Scientific, Roskilde, Denmark). Subsequently, 50 μL of substrate from the LDH assay kit was added to each well. Absorbance was read at 490 nm every minute for 30 min using a FLUOstar Omega Microplate reader (BMG LABTECH, Ortenberg, Germany).

The number of cells adhered to the Ti and polymer-coated samples were quantified using a NucleoCounter® NC-100TM (ChemoMetec A/S, Lillerød, Denmark) system accordingly with the manufacturer's guidelines. In short, 50 μL of NucleoCounter® lysis buffer was added directly onto the samples, following by vortexing. Subsequently, 50 μL of stabilisation buffer was added. This cell suspension was then loaded into a Nucleocassette™, which had been pre-coated with fluorescent propidium iodide, which stains cell nuclei. In addition, the quantity of cells in suspension was also determined using the same NucleoCounter® system to ensure a thorough analysis of cell adhesion and suspension populations.

To evaluate the cell morphology and adherence of the cells onto the Ti and polymer-coated samples, THP-1 cells that had adhered to the materials were fixed for 15 min using a 4 % formaldehyde solution (HistoLab AB, Askim, Sweden), followed by two washes with PBS. Subsequently, the cells adhered to the materials were stained with phalloidin (ActinRed™ 555 ReadyProbes™ Reagent, Rhodamine phalloidin, Invitrogen, Waltham, MA, USA), which selectively binds to F-actin and serves as a marker for total adhered cells. Nuclei were stained with DAPI (Ibidi, Fitchburg, WI, USA) before imaging using a Nikon C2+ confocal laser-scanning microscope (CLSM; Nikon, Tokyo, Japan) equipped with a 20 × /0.75 NA objective.

### 2.14 Confocal laser scanning microscopy of *S. aureus* 132_GFP_ biofilms on AgSD containing polymers

One mL of *S. aureus* 132_GFP_ (OD_600_ = 0.1) inoculum, prepared as described above was added to polymer spin-coated Ti discs containing 1 % AgSD in 24-well plates and were incubated statically at 37 °C for 4 h and 24 h, respectively. Following this, discs were gently rinsed in sterile saline to remove non-adherent bacteria and stained with PI (FilmTracer) in saline for 20 min protected from light. Following staining, discs were gently rinsed once more in saline (0.9 %) to remove unbound stain and imaged using a Nikon C2+ confocal laser scanning microscope (Nikon, Tokyo, Japan) equipped with a 100 × /1.10 NA water dipping objective (CFI Plan 100 XC W). Biofilm z-stack images were taken at 3 μm slices throughout and maximum intensity projections are presented. Two independent biological experiments were performed (n = 2) with two technical replicates.

## 3 Results

### 3.1 Material characterisation

For this study, Ti6Al4V discs (Grade V) were used as material substrate. Polymers PDLLA (P), PDLLA-mPEG (80:20) (PP20), and PDLLA-mPEG (60:40) (PP40) were synthesised to develop an antibiofilm coating to protect Ti6Al4V from bacterial colonisation. Both P and PP20 were successfully coated directly onto Ti, however, due to the rapid hydration and release of the PP40 coating from Ti, a gradient multilayer (M) was produced, improving the relative attachment to Ti (Fig. 1A–C). The addition of hydrophilic mPEG_5000_ into the polymer chain increased the hydrophilicity of the coatings, with measurements taken after 0 and 60 s to see how the contact angle changed over time (Fig. 1B). Ti6Al4V was the most hydrophobic surface and showed the highest contact angle (98.7°), with the addition of the polymeric coatings PDLLA, PP20, PP40, Multilayer and mPEG, the contact angle decreased to 63.6°, 54.0°, 38.5°, 32.5°, and 23.9°, respectively. Between 0 and 60 s, the greatest evolution in contact angle was seen in the PP40 and M surfaces, with a decrease of ∼50 % after 60 s of water droplet contact with the coating (Fig. 1B). The resulting surface topography and coating homogeneity was analysed through SEM to determine the integrity of the coatings after the spin coating procedure. The bare Ti6Al4V showed an irregular topography, meanwhile all the other materials showed a homogeneously applied coating on the surface of Ti6Al4V (Fig. 1D). Through AFM, the topography, filling capabilities and surface roughness were analysed, showing that Ti6Al4V had a comparatively complex topography and higher roughness (247 nm), while the PEGylated coated materials had a better filling capability covering >69 % of the surface area (Fig. 1E and F). This filling effect reduces surface roughness after coating application, with the polymer coated materials exhibiting a roughness between 26 and 80 nm (Fig. 1F). A summary table of the material characterisation for Ti6Al4V, homopolymers and copolymers is provided in Fig. 1F to fully describe the material surface properties (Fig. 1F).

### 3.2 Protein adsorption and cytocompatibility of polymer coatings towards THP-1 macrophages

**Fig. 2:**
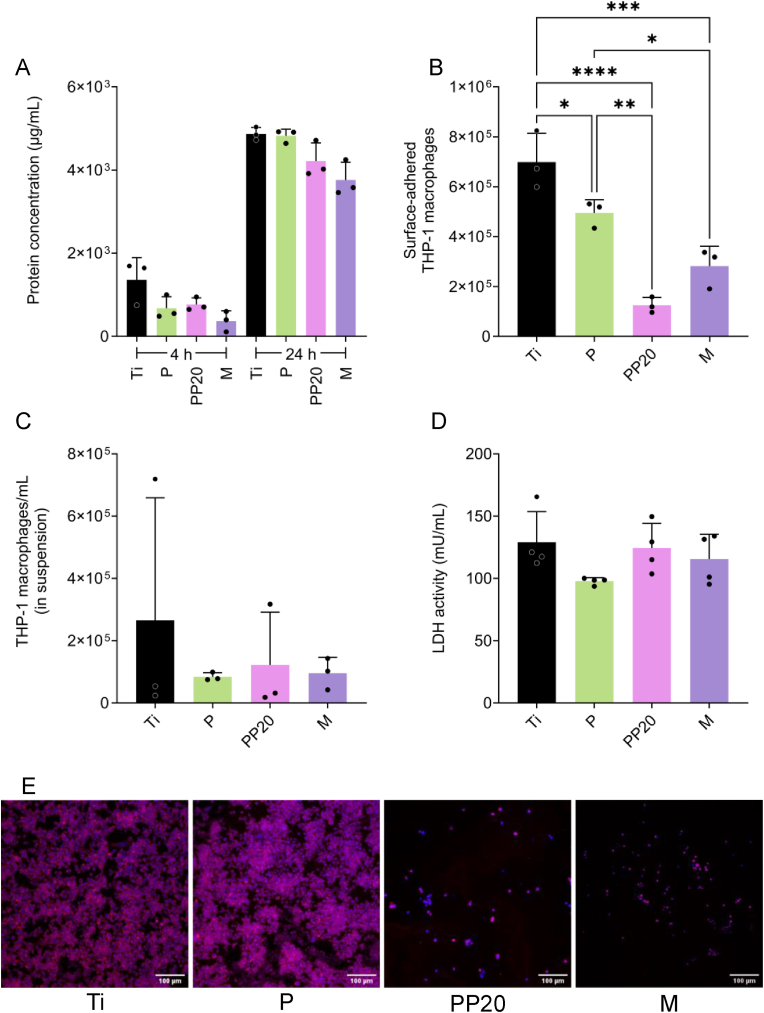
Protein adsorption of heat-inactivated foetal bovine serum (HI-FBS), and adhesion and viability of THP-1 macrophages on polymer-coated titanium. **A)** HI-FBS protein adsorption to the Ti control and polymer-coated Ti, after 4- and 24 h. **B)** Total THP-1 macrophages adhered or **C)** in suspension when cultured on the polymer-coated Ti. **D)** Cytotoxicity of the polymer coatings identified by cell membrane leakage of LDH into the culture media. **E)** Representative confocal micrographs of surface-adhered THP-1 cells on the Ti and polymer-coated Ti. Blue = DAPI/Nuclei; Red = ActinRed555/Cytoskeleton. Scale bar = 100 μm. Data represent the mean ± SD of three (**A-D:** n = 3) or four (**E:** n = 4) independent biological experiments and were analysed with one-way ANOVA followed by Tukey post-hoc test, with a *p* < 0.05 considered significant (∗P ≤ 0.05, ∗∗P ≤ 0.01, ∗∗∗P ≤ 0.001, ∗∗∗∗P ≤ 0.0001). **Abbreviations:** Polyethylene glycol (PEG), Poly-D,L-lactide (PDLLA), PDLLA-PEG 80:20 (PP20), Multilayer (M), Lactate dehydrogenase (LDH). (For interpretation of the references to colour in this figure legend, the reader is referred to the Web version of this article.)

To assess the application of these different polymers as an antimicrobial strategy for medical devices, their cytocompatibility was assessed by measuring their ability to adsorb serum proteins and their interaction with host immune cells. The adsorption of serum (HI-FBS) proteins to the Ti, P, and PP20 surfaces was comparable (Fig. 2A), whereas the M coating showed a significant reduction in the adsorption of HI-FBS proteins at 4 and 24 h (Fig. 2A). All polymer coatings showed a reduced number of surface-adhered THP-1 macrophages compared with the Control-Ti (Fig. 2B). However, the number of macrophages adhered to the polymer coatings remained high, ranging from 1.2 × 10^5^ to 4.9 × 10^5^ cells/surface (Fig. 2B), with comparable macrophage numbers in suspension for all material groups (Fig. 2C). Furthermore, all evaluated polymer coatings were cytocompatible, as demonstrated by the low LDH values, which were comparable to those from the control-Ti (Fig. 2D). The observation that the Ti and P had a higher number of surface-adhered macrophages compared to the PEGylated co-polymers PP20 and M was confirmed by CLSM (Fig. 2E).

### 3.3 Role of polymer coating on *S. aureus* 132 viability and biofilm morphology

**Fig. 3:**
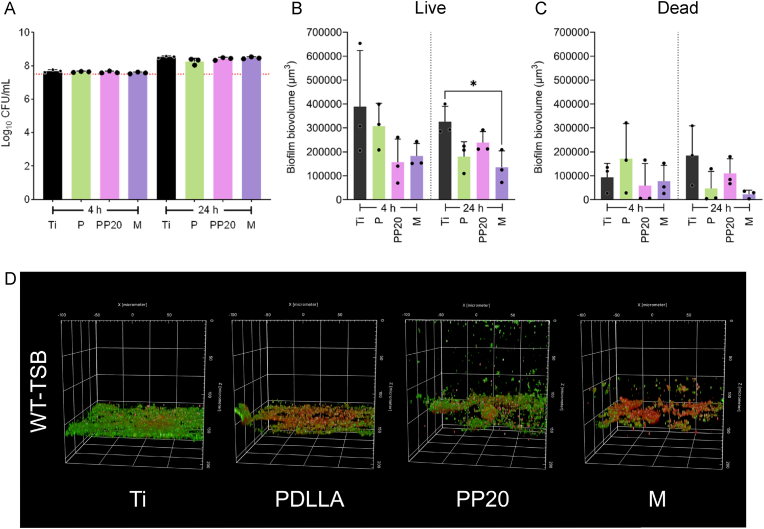
*Staphylococcus aureus* 132_GFP_ biovolume of live (green) and dead (red) cells adhered to Ti and polymer-coated Ti after 4- and 24 h. **A)***S. aureus* 132_GFP_ viable colonies after 4 h and 24 h of growth on the material surfaces. **B)** Live (GFP-positive) and **C)** Dead (PI-positive) *S. aureus* 132_GFP_ adhered to the surface of the Ti and polymer-coated Ti conditions, after 4 h and 24 h, by quantitative high-content screening confocal image analysis. **D)** Representative 3D confocal micrographs of *S. aureus* 132_GFP_ biofilms grown in TSB on each of the respective surfaces after 24 h. Data represent the mean ± SD of three independent biological experiments (n = 3). Data were analysed with one-way ANOVA followed by Tukey post-hoc test with a *p* < 0.05 considered significant (∗P ≤ 0.05). **Abbreviations:** Polyethylene glycol (PEG), Poly-D,L-lactide (PDLLA), PDLLA-PEG 80:20 (PP20), Multilayer (M). (For interpretation of the references to colour in this figure legend, the reader is referred to the Web version of this article.)

**Fig. 4:**
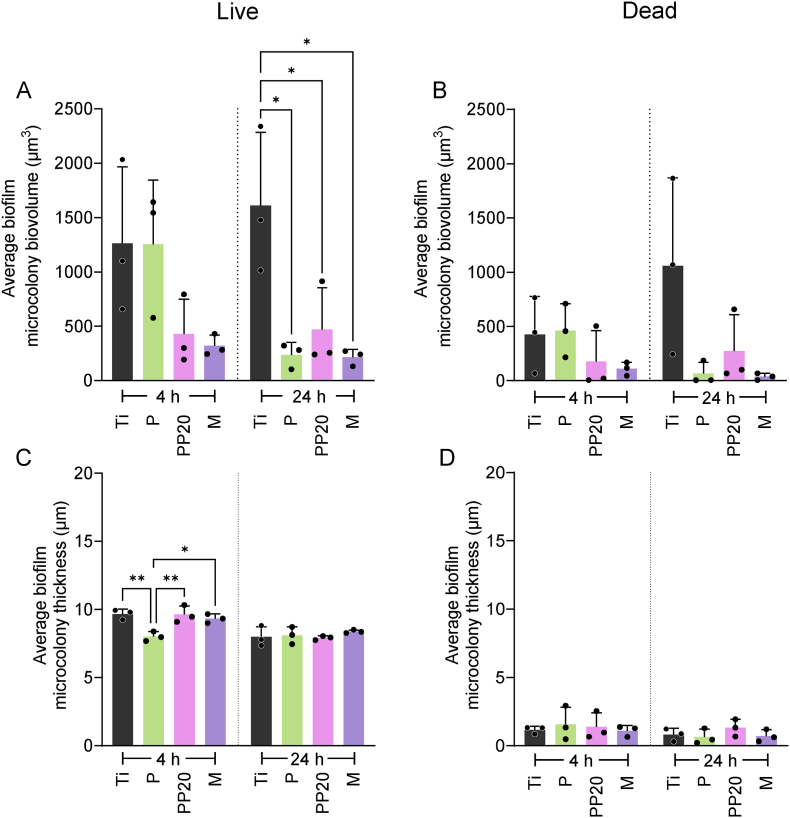
*Staphylococcus aureus* 132_GFP_ microcolony composition in biofilms adhered to control-Ti and P, PP20, and M-coated Ti after 4 h and 24 h. **A)** Average Live (GFP-positive) and **B)** Dead (PI-positive) microcolony biovolume after 4 and 24 h growth on all material surfaces. **C)** Corresponding Live (GFP-positive) and **D)** Dead (PI-positive) microcolony height, after 4- and 24 h growth, on all material surfaces. Data represent the mean ± SD of three independent biological experiments (n = 3). Data were analysed with one-way ANOVA followed by Tukey post-hoc test with a *p* < 0.05 considered significant (∗P ≤ 0.05, ∗∗P ≤ 0.01). **Abbreviations:** Polyethylene glycol (PEG), Poly-D,L-lactide (PDLLA), PDLLA-PEG 80:20 (PP20), Multilayer (M), Propidium Iodide (PI).

To assess the potential bactericidal effects of the coatings on *S. aureus* 132_GFP_, viable colony counting was employed. None of the polymer coatings exhibited bactericidal effects against *S. aureus*, with similar viable CFU counts on all polymer coatings and Ti (Fig. 3A). Biofilm morphology and distribution of live (green) and dead (red) *S. aureus* cells in contact with all materials was studied through high-content screening quantitative confocal microscopy. A decreasing trend in biofilm biovolume of live *S. aureus* 132_GFP_ cells was observed for the PEGylated polymer coatings (PP20 and M) at 4 h and 24 h, with significantly less biofilm biovolume on the M at 24 h (Fig. 3B). No changes in the dead biofilm biovolume were observed, confirming the coatings did not exert a bactericidal effect (Fig. 3C). Representative 3D-biofilm Z-stacks from each of the materials show the swelling of the polymer coating and decreasing biofilm biovolume of live bacterial cells on the PP20 and M coatings (Fig. 3D). A lower average biofilm microcolony biovolume was observed on PP20 and M at 4 h (Fig. 4A), which was significantly reduced on all three polymer coatings (P, PP20, M) compared to Ti at 24 h (Fig. 4A). The dead microcolony biovolume remained equivalent on all material surfaces (Fig. 4B). In addition, the thickness of the live biofilm microcolonies was significantly reduced on the P coating at 4 h but not at 24 h (Fig. 4C). The thickness of the dead biofilm microcolonies was equivalent on all materials (Fig. 4D).

### 3.4 Role of biofilm EPS composition on the antibiofilm properties of polymer-coated Ti

*S. aureus* biofilms are complex structures and the components of their extracellular polymeric substances (EPS) play fundamental roles in intercellular adhesion and colonisation of material surfaces. Given that the *S. aureus* strain 132 can be induced to form biofilms with EPS predominantly composed of either polysaccharides or proteins depending on environmental conditions [16], we investigated whether these polymer coatings had similar effects on these different biofilms. *S. aureus* 132 was grown in TSB_G_ to produce a proteinaceous biofilm matrix and in TSB_N_ to produce an exopolysaccharidic biofilm matrix.

**Fig. 5:**
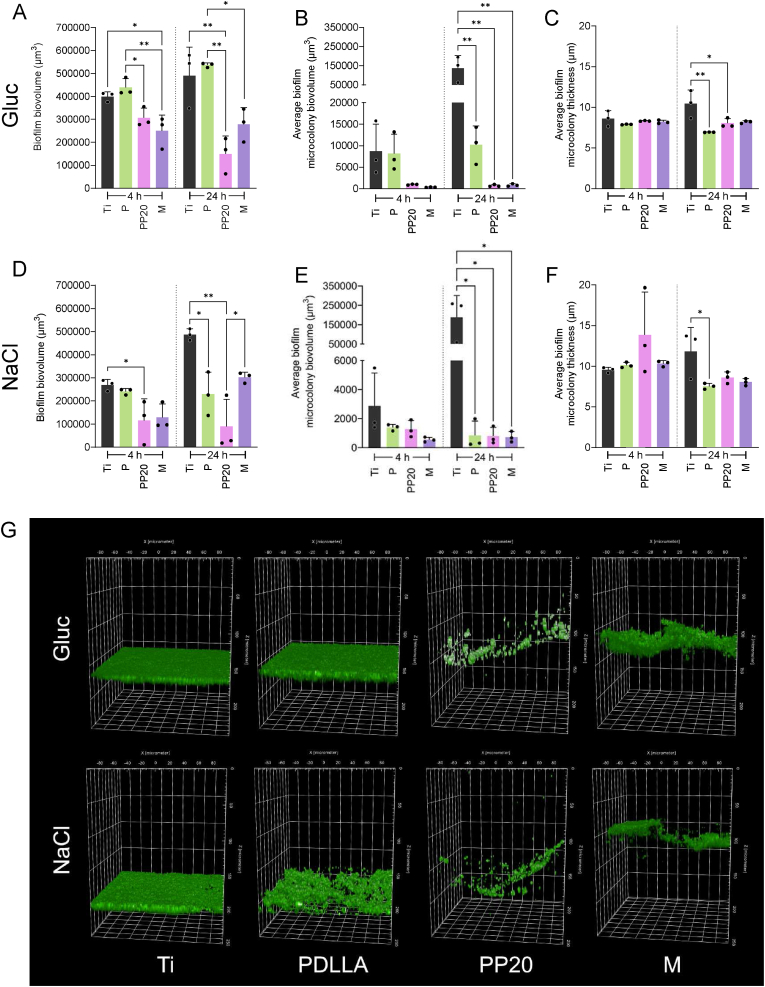
Biofilm formation of *S. aureus* 132_GFP_ on Control-Ti and Polymer-coated Ti under environmental induction conditions (Gluc (0.25 %) or NaCl (3 %)) after 4 h and 24 h. **A)***S. aureus* 132_GFP_ biofilm biovolume grown in Gluc (0.25 %) adhered to the Control-Ti and Polymer-coated Ti. **B)** Corresponding constituent microcolonies contained within the biofilm and the respective **C)** microcolony height. **D)***S. aureus* 132_GFP_ biofilm biovolume grown in NaCl (3 %) for 4 h and 24 h adhered to the Control-Ti and Polymer-coated Ti. **E)** Corresponding constituent microcolonies contained within the biofilm and the respective **F)** microcolony height. **G)** Representative 3D-confocal micrographs of *S. aureus* 132_GFP_ induced biofilms in Gluc and NaCl grown on each of the material surfaces for 24 h. Data represent the mean ± SD of three independent biological experiments (n = 3). Data were analysed with one-way ANOVA followed by Tukey post-hoc test with a *p* < 0.05 considered significant (∗P ≤ 0.05, ∗∗P ≤ 0.01, ∗∗∗P ≤ 0.001), ∗∗∗∗P ≤ 0.0001. **Abbreviations:** Polyethylene glycol (PEG), Poly-D,L-lactide (PDLLA), PDLLA-PEG 80:20 (PP20), Multilayer (M), Propidium Iodide (PI), Glucose (Gluc), Sodium chloride (NaCl).

In glucose-induced conditions (TSB_G_), both PP20 and M coatings exhibited significantly less biofilm biovolume when compared to P at 4 and 24 h (Fig. 5A). Further, at 4 h, significantly less biofilm biovolume was found on the M compared to Ti (Fig. 5A). Interestingly, the biofilm biovolume on PP20 decreased over time, and was significantly lower than on the Ti after 24 h (Fig. 5A). Moreover, at 24 h microcolony biovolume was significantly reduced on the P, PP20, and M surfaces compared to the Ti (Fig. 5B). This was also confirmed with a decrease in microcolony height on P and PP20 after 24 h (Fig. 5C).

Under NaCl-induced conditions (TSB_N_), biofilm biovolume was reduced on both PEGylated polymer coatings, especially PP20 when compared to the P and Ti at 4 h (Fig. 5D). Interestingly, after 24 h, biofilm biovolume was reduced in all polymer groups when compared to Ti, though only significantly on P and PP20 (Fig. 5D). At 4 h, microcolony volume and thickness was comparable between groups (Fig. 5E and F), however, at 24 h, biofilm microcolony volume was significantly lower on all polymers compared to Ti (Fig. 5E). Microcolony height on P was significantly lower than Ti after 24 h (Fig. 5F). The reduction in biofilm biovolume and the biofilm disruption due to co-polymer swelling can be observed in the representative 3D confocal micrographs (Fig. 5G).

**Fig. 6:**
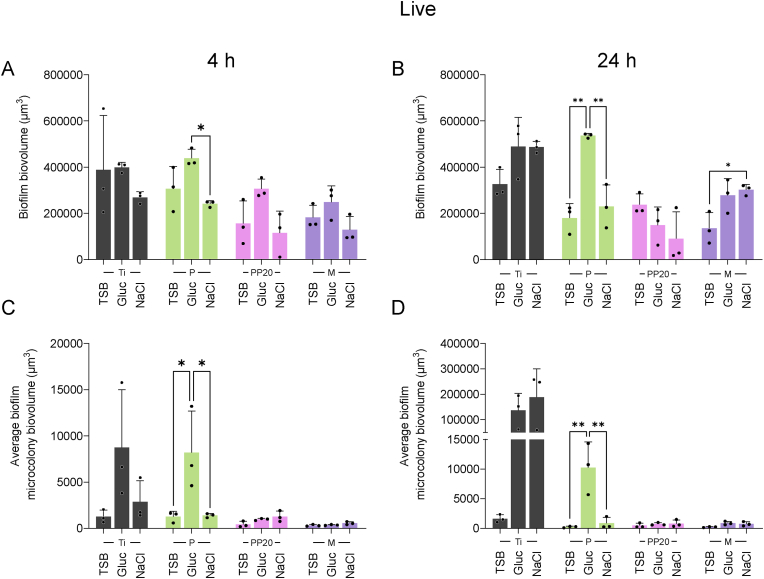
The role of media induction (TSB, TSB_G_, or TSB_N_) on *S. aureus* 132_GFP_ biofilm formation on Control-Ti and polymer coated Ti. **A)** The role of media-based proteinaceous (Gluc) and polysaccharidic (NaCl) biofilm induction on the relative levels of Live biofilm adhered to the Control-Ti and Polymer-Coated Ti after 4 h and **B)** 24 h and the respective **C)** Live microcolony biovolume adhered after 4 h and **D)** 24 h. Data represent the mean ± SD of three independent biological experiments (n = 3) and were analysed with one-way ANOVA followed by Tukey post-hoc test with a *p* < 0.05 considered significant (∗P ≤ 0.05, ∗∗P ≤ 0.01). **Abbreviations:** Polyethylene glycol (PEG), Poly-D,L-lactide (P), PDLLA-PEG 80:20 (PP20), Multilayer (M), Glucose (Gluc), Sodium chloride (NaCl).

Fig. 6 shows the difference in the formation of biofilms with different EPS compositions on each specific material surface. After 4 h, *S. aureus* 132_GFP_ wildtype biofilms formed in TSB_G_ were generally greater in biovolume than those grown in TSB and TSB_N,_ with significantly more biofilm biovolume on the P coating in TSB_G_ compared to TSB_N_ at 4 and 24 h and compared to TSB at 24 h (Fig. 6A and B). Greater biofilm biovolume was also observed on the M coating in TSB_N_ when compared to the TSB (Fig. 6B). Microcolony biovolume on P was significantly higher in TSB_G_ compared to both TSB and TSB_N_ at 4 h and 24 h (Fig. 6C and D).

### 3.5 Role of biofilm EPS composition (*S. aureus* Δ*icaADBC* and Δ*srtA*) on the antibiofilm properties of polymer-coated Ti

**Fig. 7:**
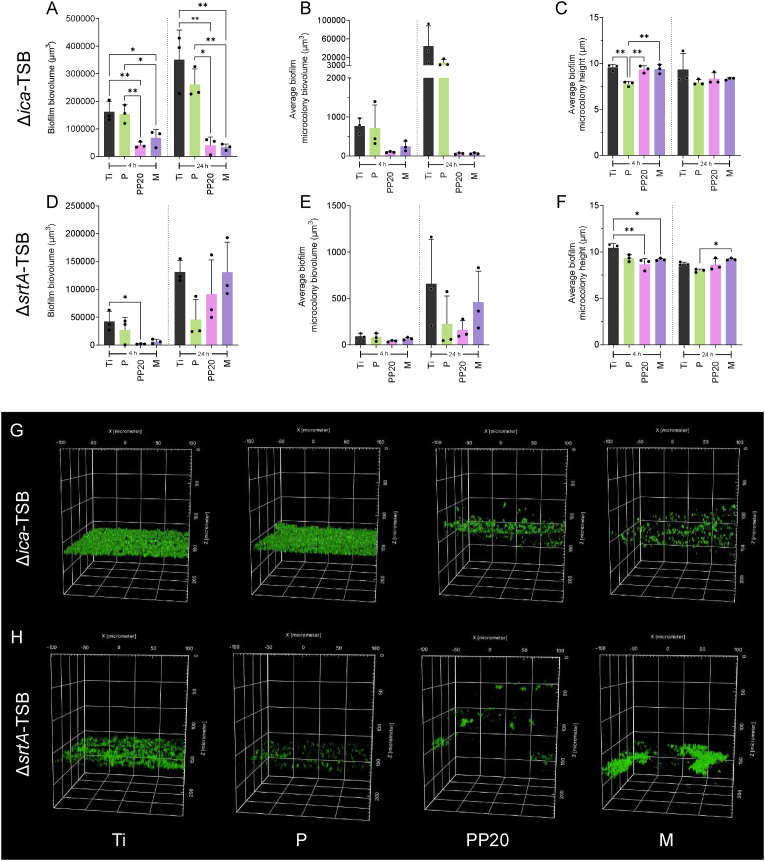
Biofilm forming abilities of *S. aureus* 132 Δ*ica*_GFP_ and Δ*srtA*_GFP_ grown in TSB on Ti and polymer-coated Ti after 4- and 24 h. **A)** Live *S. aureus* 132 Δ*ica*_GFP_ biofilm biovolume adhered to the Control-Ti and P, PP20, and M polymer-coated Ti groups after 4 h and 24 h with **B)** the average biofilm microcolony biovolume at 4 h and 24 h and the **C)** microcolony height at 4 h and 24 h. **D)** Live *S. aureus* 132 Δ*srt*A_GFP_ biofilm biovolume adhered to the Control-Ti and P, PP20, and M polymer-coated Ti groups after 4 h and 24 h with **E)** the average biofilm microcolony biovolume at 4 h and 24 h and the **F)** microcolony height at 4 h and 24 h. **G)** Representative 3D confocal micrographs of *S. aureus* 132 Δ*ica*_GFP_ and **H)***S. aureus* 132 Δ*srtA*_GFP_ biofilms grown on the respective groups after 24 h. Data represent the mean ± SD of three independent biological experiments (n = 3). Data were analysed with one-way ANOVA followed by Tukey post-hoc test, with a *p* < 0.05 considered significant (∗P ≤ 0.05, ∗∗P ≤ 0.01). **Abbreviations:** Polyethylene glycol (PEG), Poly-D,L-lactide (P), PDLLA-PEG 80:20 (PP20), Multilayer (M), Propidium Iodide (PI).

To confirm if the antibiofilm mechanism of the polymer coatings was associated with a specific biofilm EPS composition, the materials were inoculated with isogenic mutants lacking the ability to synthesise PIA (Δ*icaADBC***)** or MSCRAMMs (Δ*srtA*). After 4 h, *S. aureus* 132 Δ*ica*_GFP_ adhesion to the PEGylated co-polymers (PP20 and M) was significantly reduced compared to P and Ti (Fig. 7A). While biofilm biovolume on P and Ti increased over time, biovolume on the PP20 and M remained similar (Fig. 7A). At 24 h biofilm biovolume on PP20 and M was significantly lower than that formed on the P and Ti (Fig. 7A). This trend was also reflected at both time points in the biovolume of microcolonies (Fig. 7B). *S. aureus* 132 Δ*ica*_GFP_ microcolony height on P was significantly reduced compared to the rest of the materials at 4 h (Fig. 7C), reflecting the thinner, but denser biofilm observed by imaging (Fig. 7G).

In contrast to the large reduction in biofilm formation observed in the *S. aureus* 132 Δ*ica*_GFP_ strain, the only notable reduction in biofilm biovolume in the *S. aureus* 132 Δ*srtA*_GFP_ strain was on the PP20 at 4 h, and no differences between the surfaces were observed at 24 h (Fig. 7D). Similarly, no difference in microcolony biovolume was observed between any of the materials at either 4 h or 24 h (Fig. 7E). A reduction in microcolony height on the PP20 and M was observed at 4 h compared to Ti. However, at 24 h the microcolony height on the M was greater than on P (Fig. 7F). Fig. 7H contains representative 3D confocal images of biofilms formed by *S. aureus* 132 Δ*srtA* reflecting these observations.

**Fig. 8:**
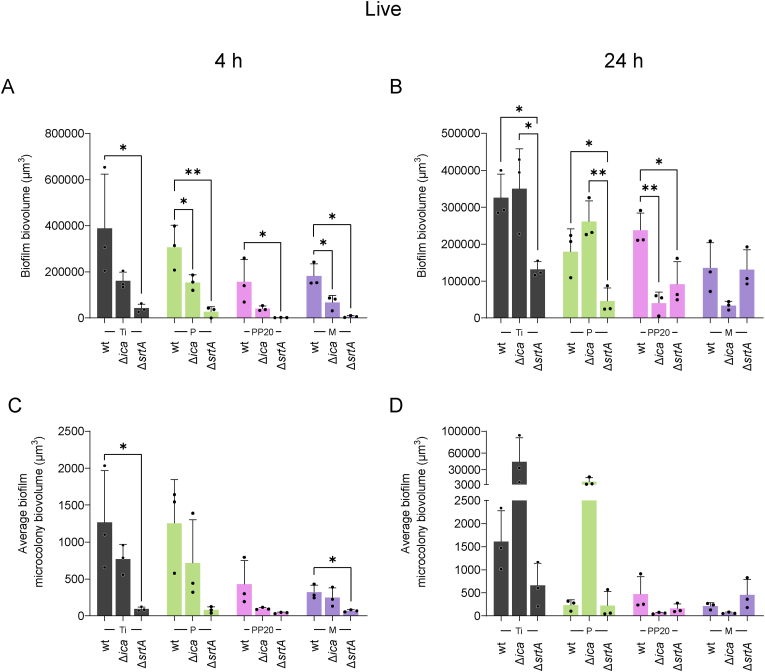
The role of polysaccharidic (Δ*ica*) and proteinaceous (Δ*srtA*) biofilm forming pathways on *S. aureus* 132_GFP_ biofilm formation on Ti, P, PP20, and polymer coated Ti. **A)** Biofilm biovolume formed by *S. aureus* 132_GFP_, *S. aureus* 132 Δ*ica*_GFP_ and *S. aureus* 132 Δ*srtA*_GFP_ after 4 h and **B)** 24 h growth in TSB on the Control-Ti, P, PP20, and M coated Ti. **C)** Average microcolony biovolume formed by *S. aureus* 132_GFP_, *S. aureus* 132 Δ*ica*_*G*FP_ and *S. aureus* 132 Δ*srtA*_GFP_ after 4 h and **D)** 24 h. Data represent the mean ± SD of three independent biological experiments (n = 3) and were analysed with one-way ANOVA followed by Tukey post-hoc test with a *p* < 0.05 considered significant (∗P ≤ 0.05, ∗∗P ≤ 0.01). **Abbreviations:** Polyethylene glycol (PEG), Poly-D,L-lactide (P), PDLLA-PEG 80:20 (PP20), Multilayer (M), Propidium Iodide (PI).

Fig. 8 shows the difference in the formation of biofilms by *S. aureus* Δ*icaADBC* and Δ*srtA* with different EPS compositions on each specific material surface. Total biofilm and microcolony biovolumes of Δ*srtA*_GFP_ were significantly reduced on all surfaces compared to the wildtype at 4 and 24 h, except on the M at 24 h (Fig. 8A-D). Regarding the *S. aureus* 132 Δ*ica*_GFP,_ after 4 h, a reduction in biofilm and microcolony biovolumes on P and M was found compared to the wildtype (Fig. 8A–C). After 24 h, biofilm biovolume produced by the *S. aureus* 132 Δ*ica*_GFP_ strain was almost identical to the wildtype on the Ti and P. However, the lack of PIA resulted in a significant reduction in biofilm formation on the PP20, and a notable reduction on the M (Fig. 8B). Microcolony volume of the Δ*ica*_GFP_ was comparable to the wildtype on all materials at 24 h (Fig. 8C and D)

### *3.6* *S. aureus* 132 biofilm antibiotic susceptibility

Biofilm formation on medical devices significantly increases the risk of treatment failure – and so, methods to improve the likelihood of treatment success are vital. To understand whether the observed disruption of biofilms formed on the polymer coatings played any role in their susceptibility to antibiotics, biofilms of different compositions formed in the Calgary biofilm device were challenged to nine antimicrobial agents. MIC characterisation of *S. aureus* 132_GFP_ confirmed phenotypic multi-resistance towards RIF, FA, CLI, OXA, and FOX (Fig. S1A).

**Fig. 9:**
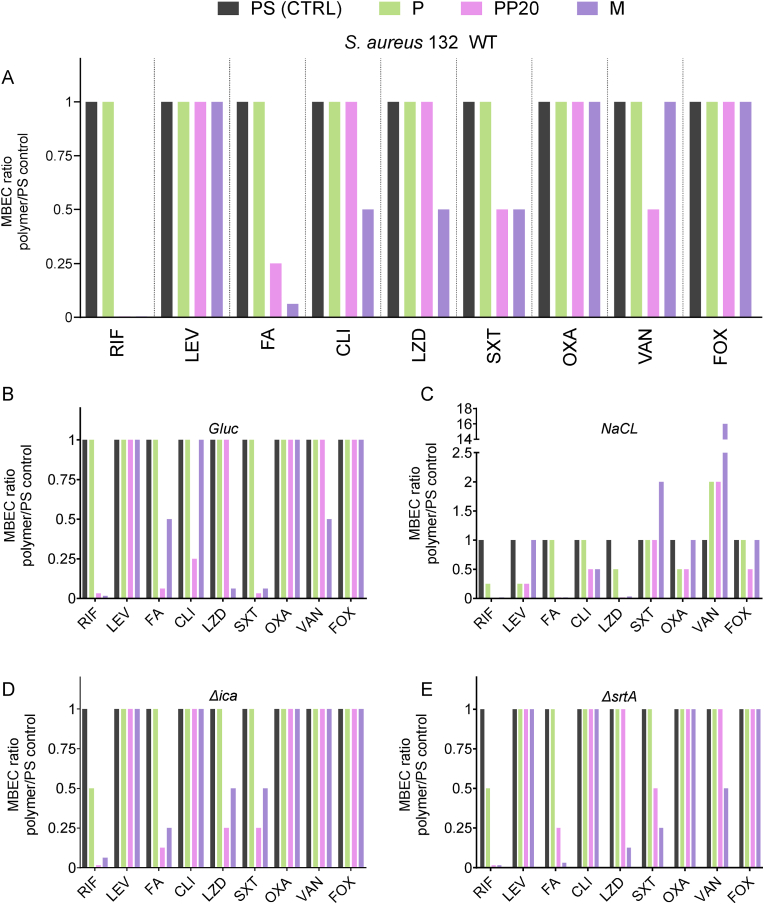
Minimum biofilm eradication concentration (MBEC) of *S. aureus* 132_GFP_ biofilms grown in **A)** TSB, **B)** TSB_G_, and **C)** TSB_N_, and **D)***S. aureus* 132 Δ*ica*_GFP_ and **E)***S. aureus* Δ*srtA*_GFP_ biofilms grown in TSB. MBEC ratios were compared against the MBEC breakpoint of the uncoated PS Calgary biofilm device peg. **Abbreviations:** Rifampicin (RIF), Levofloxacin (LEV), Fusidic acid (FA), Clindamycin (CLI), Linezolid (LZD), Sulfamethoxazole/Trimethoprim (SXT), Oxacillin (OXA), Vancomycin (VAN), Cefoxitin (FOX), Polystyrene (PS), Poly-D,L-lactide (P), PDLLA-PEG 80:20 (PP20), Multilayer (M), Glucose (Gluc), Sodium chloride (NaCl).

*S. aureus* 132_GFP_ biofilms grown in TSB were entirely resistant to all antibiotic concentrations of all tested antibiotics when biofilms were formed on the control PS pegs and on PDLLA-coated pegs (P) (Fig. 9A, Figure S1B). Biofilms grown on the PP20 and M coated pegs, however, showed increased susceptibility to four (RIF, FA, SXT, VAN) and five (RIF, FA, CLI, LZD, SXT) antibiotics, respectively (Fig. 9A). In both PP20 and M groups, susceptibility to RIF was significantly increased by > 128-fold (from >128 μg/mL to 1 μg/mL). Susceptibility to FA was also increased by > 8-fold when biofilms were grown on the M, compared to those on the P and PS (Fig. 9A).

The role of media composition (glucose and NaCl induction) on biofilm susceptibility to antibiotics was also investigated to understand if environmental modulation of biofilm composition could affect antibiotic efficacy. In glucose (TSB_G_), *S. aureus* 132_GFP_ biofilms grown on the PS control and P were entirely resistant to almost all antibiotics (Fig. 9B), with only RIF showing any antimicrobial effect (32 μg/mL) when compared to only TSB **(**Fig. S1C, B). Interestingly, biofilms grown in TSB_G_ on the PP20, and M coated pegs became more susceptible to four (RIF, FA, CLI, SXT) and five (RIF, FA, LZD, SXT, VAN) antibiotics, respectively, compared to PS and P (Fig. 9B). For *S. aureus* 132_GFP_ biofilms grown in TSB_G_, susceptibility to RIF was increased by 32- and 64-fold on the PP20 and M, respectively, compared to PS and P (Fig. 9B, and Fig. S1C, E). The susceptibility to LZD and SXT was also increased >8-fold in biofilms grown on the M coating, while biofilms on PP20 were >16-fold more susceptible to SXT (Fig. 9B and Fig. S1C).

NaCl played a role in the potentiation of three antibiotics in the PS control compared to the WT-TSB. In NaCl (TSB_N_), *S. aureus* 132_GFP_ biofilms grown on the PS control were resistant to most antibiotics (Fig. 9B), however, biofilms were more susceptible against RIF (>8-fold), SXT (>16-fold), and VAN (>64-fold) when compared to grown only in TSB **(**Figure S1C, B). In the P material, susceptibility increased in four antibiotics: RIF (4-fold), LEV (>2-fold), LZD (≥2-fold), OXA (≥2-fold), SXT (>16-fold), and VAN (>32-fold) when compared to the PS in TSB_N_, however, the MBEC for VAN increased 2-fold (Fig. S1C, B). The PP20-coated group showed the highest increase in susceptibility for six antibiotics: RIF (133-fold), LEV (>2-fold), FA (>32-fold), CLI (≥2-fold), LZD (>32-fold), and OXA (≥2-fold) compared to PS in TSB_N_, however, the MBEC for VAN increased 2-fold. Additionally, in the M group, susceptibility was increased towards four antibiotics: RIF (64-fold), FA (>32-fold), CLI (≥2-fold), and LZD (>16-fold) compared to PS in TSB_N_, however, the MBEC for VAN increased 16-fold and for SXT increased 2-fold (Fig. 9C and Fig. S1C).

Having seen the role that environmentally induced biofilms played on antimicrobial susceptibility, we wanted to probe further and understand if *S. aureus* 132, lacking the components required for proteinaceous or polysaccharidic EPS, displayed similar susceptibility profiles when grown on the polymer coatings. Similarly to the media inductions and TSB, both *S. aureus* Δ*ica* and Δ*srtA* biofilms formed on PS control and P were widely resistant to antibiotic challenge – with only rifampicin (128 or 64 μg/mL, respectively) exhibiting some increased susceptibility compared to biofilms grown in PS and P in TSB (Fig. 9D, B). The resistance profile of both *S. aureus* 132 Δ*ica* and Δ*srtA* biofilms formed on P was the same as PS, except for a two-fold decrease in RIF susceptibility (Fig. 9D, E and Fig. S1D). Both PP20 and M coatings greatly increased the susceptibility of biofilms from *S. aureus* 132 Δ*ica* and Δ*srtA* strains compared to equivalent biofilms grown on the PS control and P (Fig. 9D, E). Interestingly, the protein-mediated biofilm formed by the Δ*ica* mutant exhibited more susceptibility on the PP20 than the PIA-mediated biofilm formed by the Δ*srtA* mutant, whereas the opposite was true for M with increased susceptibility for Δ*srtA* than Δ*ica* (Fig. 9D, E and Fig. S1D). Similarly to the TSB and media induction groups, the greatest increase in biofilm susceptibility was observed for RIF by both Δ*srtA* and Δ*ica* strains on the PP20 and M coated pegs compared to PS control and P. A 64-fold increase in RIF susceptibility (from 128 μg/mL to 2 μg/mL) was seen in both Δ*srtA* and Δ*ica* strains on the PP20 compared to PS (Fig. 9D, E). For the M coating, a 64-fold increase in RIF susceptibility was seen in Δ*srtA* biofilms, while Δ*ica* biofilms were 16-fold more susceptible to RIF compared to PS (Fig. S1D). Compared to PS and P, the greatest increase in FA and LZD susceptibility was seen in Δ*ica* biofilms grown on the PP20, and in Δ*srtA* biofilms grown on M (Fig. 9D, E). Interestingly, while an increase in susceptibility was found in all strains and culture conditions on the PP20 and M-coated pegs, biofilms recovered from these surfaces (PP20 and M) also presented the highest viable colony counts (up to 3-log_10_ greater in Δ*ica* biofilms on M than on PS control (Figure S1 E).

These results combined indicate that the PEGylated co-polymers (PP20 and M) notably increased susceptibility of *S. aureus* biofilms to several clinically important antibiotics, especially a dramatic increase in susceptibility to the highly relevant antibiofilm antibiotic RIF. It also appears that environmentally induced biofilms played a greater role on potentiating biofilm susceptibility compared to the mutant strains. It also appears that biofilms induced towards primarily polysaccharidic (NaCl) and lacking MSCRAMMs adhesin proteins (Δ*srtA*) generally exhibited increased susceptibility to these antibiotics compared to their ‘protein-containing EPS’ counterparts.

### 3.7 Addition of silver sulfadiazine (AgSD) to polymer-coated Ti

**Fig. 10:**
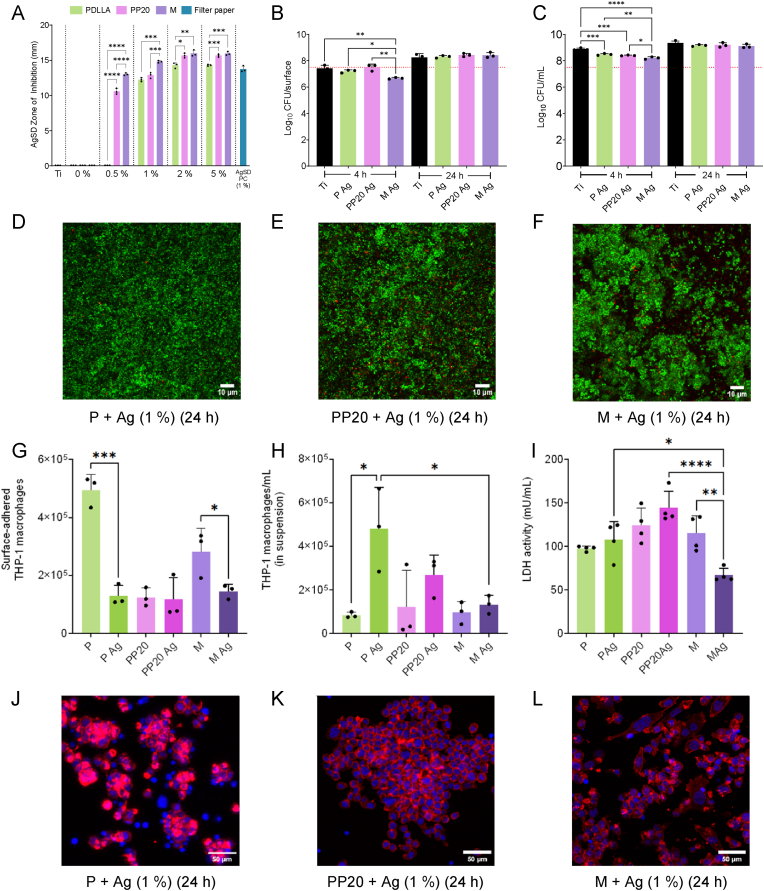
The role of AgSD addition to polymer-coated Ti on *S. aureus* 132 viability and THP-1 adhesion and viability. **A**) Antimicrobial disc diffusion diameters of AgSD against *S. aureus* 132_GFP_ on TSA at different concentrations (0–5 %) from the respective polymer coatings after 24 h. **B)** The viability of adhered *S. aureus* 132_GFP_ after 4 h and 24 h when grown on Ti and AgSD containing P, PP20, and M polymers and **C)** The viability of planktonic phase *S. aureus* 132_GFP_ surrounding the Control-Ti and AgSD containing P, PP20, and M coated Ti. The red dotted line signifies the starting inoculum CFU/mL. **D)** Maximum intensity projection of a CSLM micrograph of *S. aureus* 132_GFP_ biofilm grown for 24 h on AgSD containing P-coated Ti, **E)** PP20-coated Ti and **F)** M-coated Ti. **G)** Surface-adhered THP-1 macrophages attached to and **H)** in suspension around the P, PP20, and M coated Ti and the AgSD containing counterparts after 24 h culture. **I)** Cytotoxicity of the AgSD containing P, PP20, and M coated-Ti after 24 h culture. **J)** CLSM micrograph of THP-1 macrophages adhered to the AgSD-containing P-coated Ti, **K)** PP20-coated, and **L)** M-coated Ti after 24 h. Data represent the mean ± SD of three or four (I) independent biological experiments (n = 3 or 4) and were analysed with one-way ANOVA or followed by Tukey post-hoc test or Student's T-test (G, H, I) with a *p* < 0.05 considered significant (∗P ≤ 0.05, ∗∗P ≤ 0.01, ∗∗∗P ≤ 0.001, ∗∗∗∗P ≤ 0.0001). **D; E, F:** Green = live bacteria, red = dead bacteria, scale bar = 10 μm, **J, K, L:** Blue = DAPI/Nuclei, Red = ActinRed555/Cytoskeleton. Scale bar = 50 μm. **Abbreviations:** Polyethylene glycol (PEG), Poly-D,L-lactide (P/PDLLA), PDLLA-PEG 80:20 (PP20), Multilayer (M), Silver sulfadiazine (Ag/AgSD), Tryptic soy agar (TSA), Glucose (Gluc), Sodium chloride (NaCl). (For interpretation of the references to colour in this figure legend, the reader is referred to the Web version of this article.)

In addition to understanding how these co-polymer Ti coatings behaved in combination with clinically relevant antibiotics, we also wanted to know if direct incorporation of antimicrobial agents into the PDLLA-PEG formulation could add bactericidal effects to the observed antifouling properties. Using a modified Kirby-Bauer disc diffusion approach, we observed a concentration-dependent increase in the zone of inhibitions surrounding the AgSD blended polymers. Though these zones of inhibition surrounding the polymer groups seemed to plateau after 2 % as the diameters from all polymers remained the same at 5 %. At all tested concentrations, the addition of PEG into the polymer enhanced AgSD release as the M co-polymer group showed significantly larger zones of inhibition compared to the P – and at lower concentrations (0.5 and 1.0 %) the zones of inhibition surrounding M were also significantly larger than those from the PP20 (Fig. 10A). The elevated antimicrobial efficacy of the M co-polymer compared to the PP20, and P and the bare Ti was also present in the viable colony counting after 4 h, where biofilms cultured on the AgSD containing M were found to have significantly fewer adhered viable colonies (Fig. 10B). Substantiating the increased AgSD release from the M polymer from the disc diffusion, viable colonies from the planktonic phase surrounding the M surface were significantly lower than all other polymer groups, suggesting greater AgSD release into the surrounding media (Fig. 10C). However, in both adhered biofilms and the surrounding planktonic phase, all bactericidal effects were lost by 24 h. Similarly, the CLSM max projection shows that after 24 h, the growth and live/dead proportions of the biofilm are not hindered enough to prevent biofilm formation across all polymeric coatings (Fig. 10D-F).

As the primary function of implanted orthopaedic materials is to improve quality of life for the patient, it was also important to characterise how the addition of potentially cytotoxic agents would affect host cells and cytocompatibility of the polymer coatings. A reduction in adhered THP-1 cells compared to the equivalent polymers without AgSD was observed when AgSD was added to P and M, but no difference was seen between PP20 ± AgSD (Fig. 10G). Reflecting the decrease in adhered THP-1 cells on the P + AgSD, a significantly higher number of cells were found in suspension (Fig. 10H). Additionally, the incorporation of AgSD into the polymer coatings did not increase their cytotoxicity towards THP-1 macrophages, and interestingly in the case of the M-coated Ti, LDH levels were found to be even lower than the normal M co-polymer (Fig. 10I).

Even though no difference in the number of surface adhered THP-1 cells was observed between the AgSD containing polymer coatings, the cells were visually most homogenously dispersed in the M coated condition where cells also possessed a more stretched, spindle-like morphology. Cells on the P and PP20, however, possessed more rounded morphologies and were found to aggregate together more frequently in smaller or larger groups on the P and PP20, respectively (Fig. 10J-L).

## 4 Discussion

Here we have shown that when compared to uncoated Ti and PDLLA alone, PEG-PDLLA co-polymer coated Ti6Al4V (PP20 and M) can successfully limit, though not entirely prevent, *in vitro* biofilm formation by MRSA. Further, by inducing *S. aureus* to produce primarily protein (TSB_G_) or polysaccharidic (TSB_N_) based biofilms, the antifouling properties of the co-polymer coating were increased, confirming that the PP20 and M coatings were effective against biofilms with various EPS compositions. Moreover, the biofilm formation on these PDLLA-PEG co-polymers was more efficiently prevented when biofilm EPS lacked PIA polysaccharide (Δ*ica*) compared to when lacking MSCRAMMs surface proteins (Δ*srtA*). Most importantly, we also found that biofilms grown on these PDLLA-PEG co-polymers were notably more susceptible to a range of clinically relevant antibiotics, especially rifampicin.

Due to the cost, complexity, and limited success associated with treating orthopaedic device related infection (ODRI), prevention remains the optimal strategy for new antimicrobial strategies that aim to address this issue. Here, we investigated a bioresorbable PDLLA-PEG coating for Ti that aims to serve as a preventive approach to address ODRI. It is known that PEG reduces the surface adsorption of proteins through the combined formation of a surface hydration layer and steric repulsion, and it is thought that this reduction in protein adsorption is one of the mechanisms by which subsequent bacterial adhesion is reduced [24]. Indeed, here we found that as hydrophilicity of the polymer coatings increases (P < PP20 < M), a corresponding decrease in foetal bovine serum adsorption to the polymer surfaces was observed, as well as a decrease in biofilm formation by *S. aureus* 132 WT and Δ*ica* with functional MSCRAMMs surface adhesins. However, due to the non-specificity of the anti-adhesive properties of these coatings, it is possible to inadvertently compromise host cellular attachment or possibly even induce toxicity. To see if this occurred with the polymer coated Ti, we investigated the adhesion and cytocompatibility of THP-1 macrophages after 24 h culturing. Consistent with the decreased protein adsorption, we also observed a reduction in surface-adhered THP-1 macrophages on all surfaces compared to the Control-Ti. However, while attachment was reduced – even in the worst-case scenario ∼125 000 cells remained adhered after 24 h on the PP20. The presence of the PDLLA, PP20, or M also exhibited no deleterious effect on cell viability compared to the Control-Ti, as LDH levels were comparable between all groups after 24 h. These results align with the current literature where nanoparticles, injectables, hydrogels and films made from PDLLA and PEG showed no cytotoxic effects *in vitro* and *in vivo* [[40], [41], [42]]. While the levels of adhesion and cytocompatibility observed here are already promising, if further host-cell optimisation is desired, PEG-polymer coatings can also be combined with cell recognition sequences that overcome host-cell repulsion while maintaining the desired antifouling properties [43,44].

Following the characterisation of the host-cell response and protein adsorption to the co-polymer coatings, we wanted to understand if the polymer coatings exhibited similar anti-adhesive effects against a clinical methicillin-resistant *S. aureus* (MRSA) strain. Indeed, no effect on bacterial viability was observed after either 4 h or 24 h, further highlighting the antimicrobial role of these coatings as anti-fouling rather than bactericidal or bacteriostatic. Instead, a significant reduction in biofilm biovolume was observed on the M co-polymer after 24 h. In addition to the previously mentioned mechanisms by which PDLLA-PEG polymers prevent biofilm formation, the polymers under investigation here are bioresorbable/degradable and so undergo hydration and hydrolysis over time [27]. This hydration also contributed to the anti-biofilm effect by mechanical microcolony disaggregation and dispersal. The hydration and swelling of the polymer coatings (PP20 and M) were easily identified during confocal imaging by the greatly increased Z-distribution of *S. aureus* 132_GFP_ biofilm. The mechanical dispersal induced by the hydration and swelling was significant, since *S. aureus* 132_GFP_ microcolony biovolume was greatly reduced on both PP20 and M surfaces at 24 h. In a clinical setting, this disruption of biofilm density and reduction in microcolony size could improve microbial clearance by the host immune system. One of the limiting factors preventing adequate clearance of biofilm by the immune system is jeopardised phagocytosis resulting from the inability of phagocytes to clear such large biofilm structures [10,45]. Reducing the maturity and aggregate size of biofilms could facilitate the successful clearing of infection by immune cells, such as neutrophils and macrophages.

The MRSA strain 132 used in this study was selected for its clinical significance and because it can form either proteinaceous or polysaccharidic biofilm EPS by adding glucose or sodium chloride to the growth medium, respectively. This allows us to study, within the same genetic background, whether biofilms with specific adhesive components are affected by the polymer coatings. Indeed, when the biofilm was induced to mainly contain either PIA (TSB_N_) or protein (TSB_G_) EPS, both biofilm types were notably more disrupted by the PP20 and M. Interestingly, while less biofilm biovolume was observed in biofilms grown on the PEGylated PP20 and M compared to Ti in TSB_G_ and TSB_N_, biovolume was unchanged when compared to the equivalent WT-TSB polymer conditions, and even slightly increased on M in TSB_N_ at 24 h. Therefore, this reduction in biofilm biovolume rather reflected a relative increase in biofilm biovolume on the Control-Ti and the PDLLA induced by the TSB_G_ and TSB_N_. This suggests that with the increase in wettability, the polymer coatings, specifically the PEGylated co-polymers with mPEG_5000_, prevented this corresponding increase in biofilm biovolume. These antifouling properties have been reported for PEG with a molecular weight (MW) of 500–2000 Da in different applications [46,47]. It is also important to highlight that the molecular weight of PEG will play a critical role in cell adhesion and further optimisation is needed to properly tune the amphiphilic balance between the PDLLA and PEG.

To elucidate further the role that specific biofilm components play in the adhesion to these polymer coatings, we utilised *S. aureus* 132 Δ*icaADBC* and Δ*srtA* deletion mutants. Here, the lack of the *icaADBC* operon, and therefore PIA synthesis, significantly limited biofilm formation of both PEGylated co-polymers at 4 h and 24 h. Contrastingly, biofilm biovolume formed by *S. aureus* 132 Δ*srtA* lacking Sortase A cleaved cell wall anchored (CWA) proteins was only reduced on the polymer coatings at 4 h, as by 24 h it was equivalent on all surface types. Further, while the polymers had little effect on the *S. aureus* 132 Δ*srtA*, total biofilm biovolume was in general notably lower when compared to the WT strain in TSB, highlighting their importance of CWA/MSCRAMMs in biofilm formation. It is thought that the primary antifouling abilities of PEGylated polymers, such as the two under study here, originate from the combination of the formation of a hydration layer creating a physical and energetic barrier, and compression of the 10.13039/501100010368PEG chain generating steric repulsion [24]. Supporting this, the greatest reduction in adhesion and biofilm biovolume was observed in the strain where adhesion to the surfaces is solely or primarily mediated by the proteinaceous surface adhesins (Δ*ica*). Though, the effects observed here could be multifactorial. By lacking PIA and relying mostly on protein adhesins, which are repelled by the action of the PEGylated co-polymers, those that do attach (lacking the polysaccharide intercellular adhesin) are then unable to remain bound strongly together and are more prone to disruption by the hydration of the co-polymers, as reflected in the lower microcolony biovolume.

We wanted to investigate further whether PDLLA-PEG co-polymer coatings exhibited synergistic antibiofilm effects when combined with antibiotic treatment. We therefore challenged pre-formed biofilms from each of the studied coatings on polystyrene (PS) with a panel of 9 important antibiotics used for the treatment of periprosthetic joint infection. In all conditions but WT-TSB_N_, *S. aureus* 132 biofilms (WT-TSB, WT-TSB_G_, Δ*ica*-TSB, and Δ*srtA*-TSB) grown on the control PS and PDLLA-coated (P) were resistant to all tested antibiotic concentrations with only rifampicin exhibiting the lowest MBEC values. On the PP20 and M, however, susceptibility increased dramatically to a range of antibiotics, with the greatest increase in susceptibility observed in rifampicin and fusidic acid. The specific antibiotics to which sensitivity increased do not share many properties or mechanisms of action that could suggest specific interactions other than a lack of charge and mostly being hydrophobic. This may suggest that the increase in susceptibility could therefore be associated with the physical disruption of the biofilm. Indeed, similar conclusions have been emphasised previously, where susceptibility to antibiotic challenge was correlated with biofilm density or lack thereof [48]. It is also likely that the physical disruption/disaggregation of the respective *S. aureus* 132 biofilms was significant, as the total viable bacteria recovered from each of the co-polymer coated substrates was higher than those recovered from the PDLLA (P) or uncoated (PS).

An important consideration of coatings such as these are the final medical application, and whether the treatments would survive the demanding implantation procedure. While investigating the adhesion strength and resilience of the coating to external forces was outside the scope of this work, the spin coated polymers were strongly adhered to Ti, though mPEG_5000_ was less well adhered when applied as monolayer. This was resolved by applying a multilayer strategy (P < PP20<M) to reinforce the adherence of the layers and increase their coverage [27]. Investigating the response of the coating to the potential disruption during the surgical implantation will be important to investigate further as this may reduce the antibiofilm efficacy.

One of the advantages of PEGylated polymers is the ability to incorporate certain compounds to elicit specific biological outcomes. As mentioned, other work has shown that the addition of RGD sequences can improve host cell adhesion [43,44]. Here, we wanted to test the efficacy of loading these polymers with the hydrophobic antimicrobial agent silver sulfadiazine (AgSD) and investigate their cytocompatibility and if they improve the antimicrobial effect of antifouling surfaces. The addition of AgSD to the polymer coatings showed some significant antimicrobial properties, specifically when incorporated into the PEGylated co-polymer M. These antibacterial effects were observed in the disc diffusion and viable colony counting from both surface-adhered and surrounding planktonic *S. aureus* 132_GFP._ The bactericidal effects observed at 4 h are likely the result of a rapid ‘burst release’ of the incorporated AgSD by the M co-polymer. As such, after 24 h, any bactericidal effects generated by the remaining AgSD are overcome by the generation time of *S. aureus* 132_GFP_. The addition of AgSD to the polymers also resulted in a decrease in attachment of THP-1 macrophages to the P and M polymer coatings after 24 h. Interestingly, the inclusion of AgSD (1 %) had no effect on the viability of the THP-1 macrophages, and the M coating containing AgSD presented lower cytotoxicity than the M without AgSD. It has been reported that THP-1 cells exhibit slightly higher resistance to the effects of silver than other cell lines, which may explain this observation [49].

While the degradation kinetics of these coatings can be adjusted, those investigated here were prepared to degrade completely by 28 days - after which the antibiofilm properties would be lost. This time window would target infections that may arise during the peri-implantation and wound healing period. However, it would not address late infections originating from haematogenous seeding. Nevertheless, the utility of these coatings is the flexibility of their application. As shown, the polymers can incorporate antimicrobial agents which may extend this antibiofilm efficacy, alternatively the polymer coatings may be applied to materials with intrinsic antibacterial properties like TiNbGa alloys that would present continuing infection resistance after the polymer coating degradation [50].

In this work, we address the potential use of this coating to reduce bacterial colonisation of titanium-based orthopaedic biomaterials. *In vitro* biofilm models mimicking the orthopaedic periprosthetic environment are often carried out under static conditions, unlike for dental implants which are often carried out under flow shear stress [51]. Therefore, flow conditions were not included in the *in vitro* models throughout this study. A relatively high starting inoculum (OD_600_ 0.1 ≈ 10^7^ CFU/mL) was used to establish biofilms, which in a static *in vitro* model presents a significant challenge for non-bactericidal materials. Therefore, the antifouling properties of the PP20 and M may have been even greater in a model with dynamic media flow and lower starting inoculum. This highlights the efficacy of the coatings presented in this work and the significance of the observed reduction in bacterial adhesion and biofilm formation.

## 5 Conclusion

The PEGylated PDLLA co-polymers (PP20 and M) investigated here showed effective antibiofilm properties when challenged by a methicillin-resistant *S. aureus*, reducing adhesion to the underlying Ti. Although the coating reduced biofilm formation regardless of matrix composition, the inhibition was most pronounced in biofilms with a proteinaceous matrix. This indicates that the exopolysaccharide matrix posed a greater challenge to the PEG-PDLLA coating. Additionally, the PEGylated PDLLA co-polymers (PP20 and M) provided a cytocompatible surface for THP-1 macrophages adhesion. When combined with the bactericidal effects observed with the incorporation of AgSD into the PEG-PDLLA or by treatment with antibiotics, these findings highlight the potential of these coatings to enhance the infection resistance of titanium-based orthopaedic biomaterials.

## Funding

This research was funded by the 10.13039/501100000780European Commission within the H2020-MSCA grant agreement No. 861046 (BIOREMIA-ETN); 10.13039/501100004359Swedish Research Council (2022-00853); the Swedish state under the agreement between the Swedish government and the county councils; the ALF agreement (ALFGBG-978896); the IngaBritt and Arne Lundberg Foundation (LU2021-0048); the Hjalmar Svensson Foundation; the Doctor Felix Neuberghs Foundation; the Adlerbertska Foundation; the Area of Advance Materials of Chalmers/GU Biomaterials within the Strategic Research Area initiative launched by the Swedish government; the Spanish 10.13039/100014440Ministry of Science, Innovation and Universities grant PID2020-113494RB-I00 (Agencia Española de Investigación/10.13039/501100008530Fondo Europeo de Desarrollo Regional, European Union) to I.L.; and S.O. was funded through the Government of Ireland Postgraduate Scholarship Programme by the 10.13039/501100002081Irish Research Council (GOIPG/2023/3290).

## CRediT authorship contribution statement

**Adam Benedict Turner:** Writing – review & editing, Writing – original draft, Visualization, Validation, Methodology, Investigation, Formal analysis, Conceptualization. **David Zermeño-Pérez:** Writing – review & editing, Writing – original draft, Visualization, Validation, Methodology, Investigation, Formal analysis, Conceptualization. **Margaritha M. Mysior:** Writing – review & editing, Visualization, Validation, Methodology, Formal analysis. **Paula Milena Giraldo-Osorno:** Writing – review & editing, Visualization, Methodology, Investigation, Formal analysis. **Begoña García:** Writing – review & editing, Writing – original draft, Methodology, Investigation. **Elizabeth O'Gorman:** Writing – review & editing, Methodology, Investigation. **Shafik Oubihi:** Writing – review & editing, Methodology, Investigation. **Jeremy C. Simpson:** Writing – review & editing, Resources, Methodology. **Iñigo Lasa:** Writing – review & editing, Resources, Methodology, Funding acquisition, Conceptualization. **Tadhg Ó Cróinín:** Writing – review & editing, Supervision, Resources, Project administration, Methodology, Funding acquisition, Conceptualization. **Margarita Trobos:** Writing – review & editing, Writing – original draft, Supervision, Resources, Project administration, Methodology, Funding acquisition, Conceptualization.

## Declaration of competing interest

David Zermeño-Pérez is an employee of Ashland Specialties Ireland Ltd. The remaining authors declare that the research was conducted in the absence of any commercial or financial relationships that could be construed as a potential conflict of interest.

## Data Availability

Data will be made available on request.
