# Supplementary material for: Biofilm morphology and antibiotic susceptibility of methicillin-resistant Staphylococcus aureus (MRSA) on poly-D,L-lactide-co-poly(ethylene glycol) (PDLLA-PEG) coated titanium
Source: Biofilm. 2024 Oct 5;8:100228. doi: 10.1016/j.bioflm.2024.100228 (PMC11740804; doi:10.1016/j.bioflm.2024.100228)
Supplement: Multimedia component 1 [file mmc1.pdf]

Biofilm morphology and antibiotic susceptibility of methicillin-resistant *Staphylococcus aureus* (MRSA) on poly-D,L-lactide-*co*-poly(ethylene glycol) (PDLLA-PEG) coated titanium.

Authors: Adam Benedict Turner[1,2*], David Zermeño-Pérez[3,4*], Margaritha M. Mysior[5], Paula Milena Giraldo-Osorno[1,2], Begoña García[6], Elizabeth O'Gorman[4], Shafik Oubihi[4], Jeremy C. Simpson[5], Iñigo Lasa[6], Tadhg Ó Cróinín[4,#], Margarita Trobos[1,2,#].

*Equal contribution; #Joint last authors

Author affiliation:

[1] Department of Biomaterials, Institute of Clinical Sciences, Sahlgrenska Academy, University of Gothenburg, Sweden

[2] Centre for Antibiotic Resistance Research in Gothenburg (CARe), Gothenburg, Sweden

[3] Ashland Specialties Ireland Ltd., Mullingar, Ireland

[4] School of Biomolecular and Biomedical Science, University College Dublin, Dublin, Ireland

[5] Cell Screening Laboratory, UCD School of Biology & Environmental Science, University College Dublin, Dublin 4, Ireland

[6] Microbial Pathogenesis Laboratory. Navarrabiomed-Complejo Hospitalario de Navarra (CHN)-Universidad Pública de Navarra (UPNA), IDISNA, Pamplona, Navarra, Spain

Correspondence:

Margarita Trobos: Department of Biomaterials, Institute of Clinial Sciences, Sahlgrenska Academy, University of Gothenburg, P.O. Box 412, 405 30 Gothenburg, Sweden, margarita.trobos@biomaterials.gu.se

Tadhg Ó Cróinín: UCD School of Biomolecular and Biomedical Science, Room C140, Health Science Centre, Belfield, Dublin, tadhg.ocroinin@ucd.ie

A

	Antimicrobial agent	MIC	EUCAST
MIC (µg/mL)	rifampicin	<0.015	s
	levofloxacin	32	r
	fusidic acid	<0.5	s
	clindamycin	>256	r
	linezolid	<2	s
	trimethoprim-sulfamethoxazole	<1/19	s
	oxacillin	32	r
	vancomycin	2	s
	cefoxitin	16	r

B

	Antimicrobial agent	PS (CTRL)	P	PP20	M
MBEC (µg/mL)	rifampicin	>128	>128	1	1
	levofloxacin	>128	>128	>128	>128
	fusidic acid	>256	>256	128	32
	clindamycin	>256	>256	>256	256
	linezolid	>128	>128	>128	128
	trimethoprim-sulfamethoxazole	>62/1216	>62/1216	62/1216	62/1216
	oxacillin	>64	>64	>64	>64
	vancomycin	>128	>128	128	>128
	cefoxitin	>64	>64	>64	>64

C

	Antimicrobial agent	PS (CTRL)		P		PP20		M	
		Gluc	NaCl	Gluc	NaCl	Gluc	NaCl	Gluc	NaCl
MBEC (µg/mL)	rifampicin	32	16	32	4	1	0.12	0.5	0.25
	levofloxacin	>128	>128	>128	64	>128	64	>128	>128
	fusidic acid	>256	>256	>256	>256	32	8	256	8
	clindamycin	>256	>256	>256	>256	128	256	>256	256
	linezolid	>128	>128	>128	128	>128	4	16	8
	trimethoprim-sulfamethoxazole	>62/1216	4/76	>62/1216	4/76	4/76	4/76	8/152	8/152
	oxacillin	>64	>64	>64	64	>64	64	>64	>64
	vancomycin	>128	2	>128	4	>128	4	128	32
	cefoxitin	>64	>64	>64	>64	>64	>64	>64	>64

D

	Antimicrobial agent	PS (CTRL)		P		PP20		M	
		Δica	*ΔsrtA*	*Δica*	*ΔsrtA*	*Δica*	*ΔsrtA*	*Δica*	*ΔsrtA*
MBEC (µg/mL)	rifampicin	128	128	64	64	2	2	8	2
	levofloxacin	>128	>128	>128	>128	>128	>128	>128	>128
	fusidic acid	>256	>256	>256	>256	64	128	128	16
	clindamycin	>256	>256	>256	>256	>256	>256	>256	>256
	linezolid	>128	>128	>128	>128	64	>128	128	32
	trimethoprim-sulfamethoxazole	>62/1216	>62/1216	>62/1216	>62/1216	32/608	62/1216	62/1216	32/608
	oxacillin	>64	>64	>64	>64	>64	>64	>64	>64
	vancomycin	>128	>128	>128	>128	>128	>128	>128	128
	cefoxitin	>64	>64	>64	>64	>64	>64	>64	>64

E

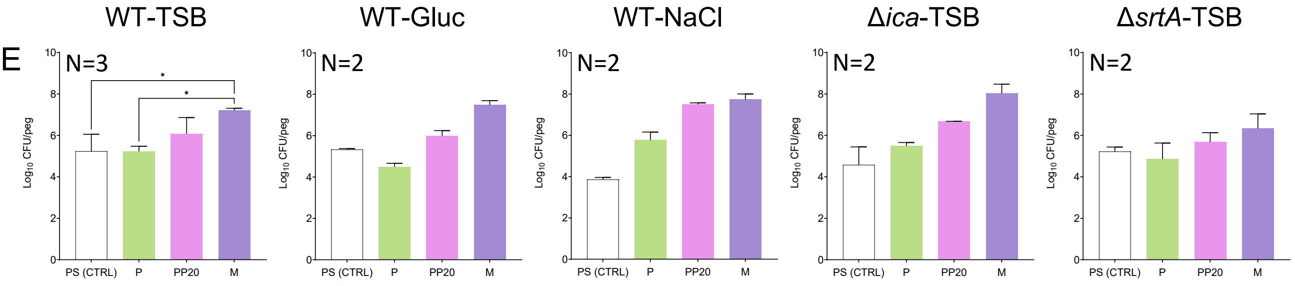

Figure S1. Antibiotic susceptibility of *S. aureus* 132 after 24 h culture on PS (control), P, PP20, and M coated Calgary biofilm pegs. **A)** Minimum inhibitory concentration (MIC) for planktonic *S. aureus* 132. **B)** Minimum biofilm eradication concentration (MBEC) for wild-type *S. aureus* 132 biofilms grown in TSB or **C)** ± Gluc/NaCl for 24 h. **D)** Minimum biofilm eradication concentrations (MBEC) for *S. aureus* 132 Δ*ica* and Δ*srtA* grown in TSB. **E)** Viable biofilms (CFU/peg) recovered from each condition and each material type. The highest common MBEC value was chosen from two (n = 2: Gluc, NaCl Δ*ica*, Δ*srtA*) or three (n = 3: WT-TSB) biological replicates. Data represent the mean ± SD from three (WT-TSB) or two (Gluc, NaCl Δ*ica*, Δ*srtA)* independent experiments. Only those with n=3 were analysed with one-way ANOVA followed by Tukey post-hoc test with a $p < 0.05$ considered significant (*P ≤ 0.05). MBEC values marked in green depict an increase in susceptibility, red depict increased resistance, and blue depict no change compared to the PS control. **Abbreviations**: Polystyrene (PS), Poly-D,L-lactide (P), PDLLA-PEG 80:20 (PP20), Multilayer (M), Glucose (Gluc), Sodium chloride (NaCl).
